# CAF to the Rescue! Potential and Challenges of Combination Antifungal Therapy for Reducing Morbidity and Mortality in Hospitalized Patients With Serious Fungal Infections

**DOI:** 10.1093/ofid/ofae646

**Published:** 2024-10-30

**Authors:** Samantha E Jacobs, Vishnu Chaturvedi

**Affiliations:** Department of Medicine, Icahn School of Medicine at Mount Sinai, New York, New York, USA; Microbiology and Molecular Biology Laboratories, Department of Pathology, Westchester Medical Center, Valhalla, New York, USA; Department of Pathology, Microbiology, and Immunology, New York Medical College, Valhalla, New York, USA

**Keywords:** aspergillosis, combination antifungal therapy, cryptococcosis, invasive candidiasis, mucormycosis

## Abstract

The global burden of invasive fungal disease is substantial and escalating. Combination antifungal therapy (CAF) may improve patient outcomes by reducing development of resistance, improving drug penetration and rate of fungal clearance, and allowing for lower and less toxic antifungal drug doses; yet, increased cost, antagonism, drug-drug interactions, and toxicity are concerns. Clinical practice guidelines recommend antifungal monotherapy, rather than CAF, for most invasive fungal diseases due to a lack of comparative randomized clinical trials. An examination of the existing body of CAF research should frame new hypotheses and determine priorities for future CAF clinical trials. We performed a systematic review of CAF clinical studies for invasive candidiasis, cryptococcosis, invasive aspergillosis, and mucormycosis. Additionally, we summarized findings from animal models of CAF and assessed laboratory methods available to evaluate CAF efficacy. Future CAF trials should be prioritized according to animal models showing improved survival and observational clinical data supporting efficacy and safety.

## Teaser

Combination antifungal therapy for invasive fungal diseases could improve patient outcomes. Prioritization of future clinical trials of combination antifungal therapy should be based on animal models showing improved survival, observational clinical data demonstrating efficacy and safety, and antifungal combination testing.

## Graphical Abstract

**Graphical Abstract:**
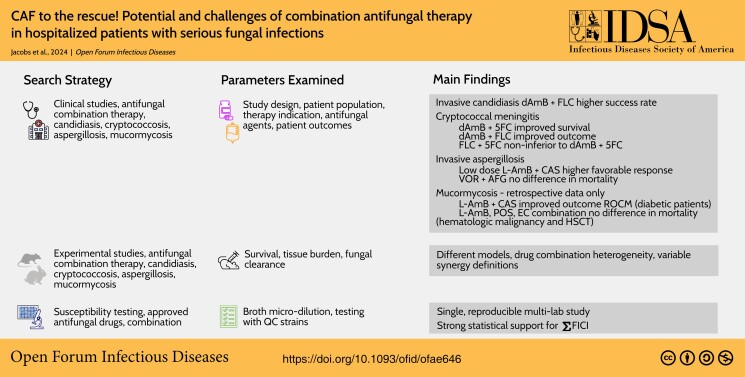
This graphical abstract is also available at Tidbit: https://tidbitapp.io/tidbits/caf-to-the-rescue-potential-and-challenges-of-combination-antifungal-therapy-for-reducing-morbidity-and-mortality-in-hospitalized-patients-with-serious-fungal-infections

## CONTINUED DISMAL OUTCOMES FOR PATIENTS WITH SERIOUS FUNGAL INFECTIONS

The global burden of invasive fungal disease (IFD) is substantial, causing approximately 1.5 million deaths per year, and it is escalating due to a growing population of individuals who are immunosuppressed, global health events such as the COVID-19 pandemic, and improvements in molecular diagnostics leading to increased reporting of previously unrecognized or undiagnosed IFD [1–6].

IFDs have considerable economic impact in terms of direct medical costs, productivity loss, and premature deaths [7]. In 2019, the estimated economic burden of fungal disease in the United States was $11.5 billion, and the mean cost of an inpatient visit associated with fungal disease diagnosis was more than twice that of the average inpatient stay. Within the health care system, IFDs lead to increased length of stay and mortality, as well as inappropriate empirical antimicrobial therapy [8–10]. Disparate mortality rates are observed across continents and countries. For example, 30-day all-cause mortality in patients with candidemia is approximately 30% in Europe and the United States and 60% to 72% in South Africa and Brazil [11].

## POTENTIAL AND CHALLENGES OF COMBINATION ANTIFUNGAL THERAPY

Given the considerable individual and public health burden of IFDs, emphasis on research pertaining to antifungal therapeutics is essential. Except for cryptococcal meningitis, current clinical practice guidelines recommend antifungal monotherapy for most IFDs; yet, morbidity and mortality remain unacceptably high [12–19]. Combination antifungal therapy (CAF) is one putative strategy to reduce risk and improve outcomes. Potential reasons to pursue CAF for IFDs are (1) to broaden the spectrum of activity for empirical therapy, (2) to enhance the rate of killing through antifungal synergy/increased potency, (3) to delay or prevent development of resistance, (4) to reduce antifungal toxicity by using lower drug dosages, (5) to enhance the effectiveness of older antifungals, (6) to treat breakthrough infections, and (7) to provide a temporary bridge to therapeutic voriconazole (VOR) levels in specific populations [20–24].

CAF may be particularly useful in difficult clinical situations (eg, infections due to highly resistant pathogens or biofilm formation) or in tissues with compromised drug penetration. However, some potentially harmful effects of CAF include reduced fungal killing (antagonism), drug-drug interactions, increased toxicity, and higher cost.

The concept of combining drugs with different mechanisms of action to treat a disease is a long-standing practice supported by rigorous randomized controlled trials (RCTs) for other complex and severe infections, including HIV and tuberculosis, as well as oncologic disease [25–28]. Unfortunately, multiple challenges to conducting large-scale RCTs exist for patients with IFDs, such as cost, the significant time to conduct the study (particularly for rare fungi; eg, Mucorales), and difficulty enrolling patients who are medically complex and/or critically ill [29]. Due to a lack of RCTs comparing CAF with monotherapy, CAF is reserved for salvage therapy or specific severe disease manifestations (eg, *Candida* endocarditis) in US and international guidelines [13].

To frame new hypotheses and carefully inform clinical trial design, it is important to examine and build on the existing body of preclinical research and human studies. We performed a systematic review of CAF clinical studies for the most prevalent and dangerous fungal pathogens: *Candida* spp, *Cryptococcus neoformans*, *Aspergillus* spp, and Mucorales. We summarized findings from experimental research and assessed laboratory methods available to evaluate CAF efficacy. Table 1 provides a qualitative summary of the evidence for CAF from a “bird’s-eye view.”

**Table 1.** Bird's-eye View: Clinical and Experimental Evidence Supporting Combination Antifungal Therapy for the Most Prevalent and Dangerous Fungal Pathogens

Disease	Qualitative Evidence Summary
Invasive candidiasis	Single RCT of FLC vs FLC + dAmB published in 2003 trended toward improved success in combination arm Experimental models of systemic *C albicans* and *C glabrata* infection primarily support polyene-echinocandin combination Paucity of experimental and clinical data investigating CAF for azole- and echinocandin-resistant *Candida* spp and *C auris* No prospective trials incorporating echinocandins for CAF have been conducted
Cryptococcosis	Multiple large RCTs in HIV/AIDS-associated cryptococcal meningitis demonstrate that induction CAF with AmB + 5-FC leads to improved microbiologic and clinical outcomes vs monotherapy Single-dose L-AmB (10 mg/kg) + 14 d × 5-FC and FLC now first-line regimen in resource-limited settings No prospective trials in non-HIV/AIDS immunocompromised hosts or in non-CNS manifestations of cryptococcosis
Invasive aspergillosis	Single large multination RCT of VOR + AFG vs VOR alone trended toward improved 6-wk mortality in hematologic malignancy cases with invasive aspergillosis (*P* = .087) Azole-echinocandin combinations strongly supported by experimental models of invasive aspergillosis Experimental studies of azole-polyene combinations primarily demonstrate antagonism; very few clinical data show no difference vs AmB alone Prospective clinical data to inform CAF for azole-resistant *Aspergillus* spp are lacking
Mucormycosis	Mixed results of azole-polyene CAF from experimental data; polyene-echinocandin CAF in vivo synergy may be species specific No prospective trials of CAF exist; largest retrospective analysis of CAF, including AmB, POS, ISA, and echinocandins, found similar 6-wk survival vs monotherapy

Abbreviations: 5-FC, flucytosine; AFG, anidulafungin; AmB, amphotericin B; CAF, combination antifungal therapy; CNS, central nervous system; dAmB, deoxycholate amphotericin B; FLC, fluconazole; ISA, isavuconazole; L-AmB, liposomal amphotericin B; POS, posaconazole; RCT, randomized controlled trial; VOR, voriconazole.

## CLINICAL STUDIES OF CAF

### Systematic Review Methods

A systematic review to identify clinical studies of CAF for invasive candidiasis (IC), cryptococcosis, invasive aspergillosis (IA), and invasive mucormycosis was conducted in accordance with the PRISMA guidelines [30]. PubMed was searched for English-language publications with the following terms: “antifungal combination therapy” or “combination antifungal therapy” and “candida” or “candidiasis,” “cryptococcus” or “cryptococcosis” or “cryptococcal,” “aspergillus” or “aspergillosis,” and “mucorales” or “mucormycosis” or “zygomycetes” or “zygomycosis” (Supplementary Appendix). We limited our search to articles published between 1 January 2003 (coinciding with the year of US Food and Drug Administration approval of VOR) and 1 February 2023. Reference lists from the included studies were additionally screened to identify potentially relevant evidence. We included studies whose primary aim was to evaluate the CAF efficacy for proven or probable IFD according to consensus definitions; fungal speciation was not required [31, 32]. Articles were excluded if they were literature reviews or meta-analyses, if they did not specify antifungal drug dosages, or if they evaluated combinations including nonsystemic antifungal agents (eg, topical or aerosolized formulations) or nonantifungal chemical compounds.

### Systematic Review Results

The PRISMA flowcharts in the Supplementary Appendix provide a detailed breakdown of the results of the primary search for each of the 4 IFDs. From 954 publications evaluated, 104 CAF articles met the inclusion criteria: 21 for IC, 17 for cryptococcosis, 45 for IA, and 21 for mucormycosis. In what follows, we focus our discussion on the findings from randomized trials and large observational studies for each pathogen. Details of each article, including study design, patient population, antifungal therapy, and patient-related outcomes, are provided in Supplementary Tables 1 to 4.

#### Invasive Candidiasis

Only 1 RCT of CAF for IC was identified in the systematic review, and the trial was conducted prior to the availability of echinocandins. The trial included 219 patients who were nonneutropenic and received fluconazole (FLC; 800 mg/d) plus placebo vs FLC plus deoxycholate amphotericin B (dAmB; 0.7 mg/kg/d) for candidemia (*Candida albicans* in 62%) [33]. There was no difference between groups in the primary analysis of time to failure or in mortality, although overall success rates were higher in the combination vs monotherapy arm (69% vs 56%, *P* = .043). Importantly, this study demonstrated that combined FLC plus amphotericin B (AmB) was not antagonistic as compared with FLC alone.

Further clinical data pertaining to CAF for IC were limited to 20 case reports and small case series, most often describing azole-echinocandin or polyene-echinocandin combinations used as salvage therapy for bloodstream infection and deep-seated infections (endocarditis, peritonitis, and septic arthritis). In the majority of case reports, source control via catheter removal or surgical debridement was performed simultaneously or following sterilization of cultures. Four case reports and a series of 13 patients with peritoneal dialysis-associated *Candida* peritonitis described favorable outcomes in patients treated with combination dAmB or liposomal AmB (L-AmB) plus flucytosine (5-FC), a regimen still recommended for treatment of deep-seated *Candida* infections (eg, meningitis and endocarditis) based on limited experimental and clinical data [13, 34–38]. This combination has shown synergy in vitro and is widely used for cryptococcal meningitis [39]. Moreover, excellent 5-FC levels are achieved in cerebrospinal fluid [40].

The paucity of controlled studies of CAF for IC precludes a determination of its safety and efficacy; yet, acquired antifungal resistance during monotherapy is well documented [41–43]. Initial CAF may mitigate development of resistance and optimize drug penetration to certain body sites. The single RCT of CAF from the past 20 years utilized dAmB, and no studies have investigated agents with more favorable safety profiles. However, as compared to rare IFDs, randomized trials of antifungal combinations for treatment of candidemia are more feasible, as evidenced by prior well-done RCTs comparing single drugs [44, 45]. To inform the design of clinical trials of CAF for IC, additional preclinical studies are needed to investigate combinations of safe agents, whether currently approved or investigational.

#### Cryptococcosis

The systematic review identified 12 RCTs and 1 prospective nonrandomized trial of induction therapy for cryptococcal meningitis, all in individuals with HIV/AIDS, as well as 3 retrospective studies and a case report in persons with other immunocompromising conditions. There are no prospective trials in persons without HIV, nor has CAF been evaluated for other manifestations of cryptococcosis (eg, pulmonary disease).

Several trials established that the combinations of AmB plus 5-FC and AmB plus FLC achieve improved clinical outcomes as compared with AmB alone [46–48]. However, fungicidal activity, as measured by the rate of reduction in cerebrospinal fluid yeast colony-forming units from serial quantitative cultures, is greater with AmB plus 5-FC as compared with AmB plus FLC [46, 47].

Combination 5-FC and FLC is appealing due to its oral availability and low rates of nephrotoxicity, although prolonged 5-FC is associated with leukopenia [49]. In a pivotal trial in Africa (ACTA) including 721 patients, the all-oral induction regimen of FLC (1200 mg/d) plus 5-FC (100 mg/kg/d) was noninferior to dAmB plus FLC or 5-FC for 7 or 14 days for the primary end point of 2-week mortality [50]. However, faster cerebrospinal fluid clearance was observed with AmB plus 5-FC when compared with FLC plus 5-FC.

L-AmB was investigated in a landmark trial, Ambition, in 844 individuals with cryptococcal meningitis in sub-Saharan Africa. A single 10-mg/kg dose of L-AmB plus 14 days of high-dose FLC and 5-FC was noninferior to the World Health Organization–recommended 1-week regimen of dAmB plus 5-FC and was associated with fewer adverse events [51]. Following the results of the ACTA and Ambition trials, as well as advocacy efforts, generic 5-FC is more widely available at a reduced cost and utilized in routine care [52].

Three-drug combinations of AmB, 5-FC, and FLC have also been studied. While the triple therapy had the most potent fungicidal effect in a murine model of cryptococcal meningitis, it was not more efficacious than AmB plus 5-FC in humans [46, 53].

Other triazole antifungal agents have excellent in vitro activity against *Cryptococcus* spp, and the combination of AmB and VOR had similar early fungicidal activity as compared with AmB plus 5-FC and AmB plus FLC in a small randomized trial [54, 55]. However, VOR, isavuconazole (ISA), and posaconazole (POS) have more side effects, more drug-drug interactions, and higher costs than FLC, thus limiting their utility for a disease that is most prevalent in resource-limited settings.

It is important to highlight the various factors that have enabled the execution of large-scale, rigorous RCTs of CAF for cryptococcal meningitis vs other mycoses. From a patient standpoint, patients enrolled into trials of cryptococcal meningitis therapy are treatment naive, and sensitive yet noninvasive diagnostic tools are widely available (eg, cryptococcal antigen). In addition, the development of early fungicidal activity, an easily measured surrogate end point for treatment efficacy, facilitated phase 2 studies of antifungal dose optimization and ultimately supported the rationale for single high-dose L-AmB in the Ambition trial. Efforts are ongoing to develop similar sensitive and specific fungal biomarkers to facilitate early diagnosis of other IFDs and correlate their dynamics to treatment response [56, 57].

#### Invasive Aspergillosis

Two randomized trials, 10 observational studies, and 33 case reports/series met inclusion criteria in the systematic review of CAF for IA.

Prior to widespread use of VOR as standard of care, prospective studies primarily investigated polyene-echinocandin combinations. In the randomized open-label Combistrat trial including 30 patients with hematologic malignancy and IA, those receiving L-AmB (3 mg/kg/d) plus caspofungin (CAS) had a higher favorable response rate (67%) and less acute kidney injury than those receiving high-dose L-AmB alone (10 mg/kg/d; 27%, *P* = .028 for comparison) for primary therapy [58]. However, L-AmB ≥7.5 mg/kg/d plus CAS led to poorer clinical outcomes as compared with POS suspension for salvage therapy in a single-center compassionate use trial [59]. Further clinical trials with polyene-based combination therapy have not been pursued in the past 2 decades given a lack of compelling preclinical data, high rates of AmB-associated nephrotoxicity, and studies confirming improved IA-related mortality with VOR [60, 61].

Polyene-triazole combination therapy has received relatively little study in humans given the preclinical data demonstrating variable synergistic interactions [62]. The only comparative data are from a retrospective study in which similar 12-week survival was observed among 49 patients receiving L-AmB (3 mg/kg/d) plus VOR or POS vs L-AmB plus CAS vs VOR plus echinocandin [63].

Observational studies of echinocandin-triazole combination therapy for IA have had inconsistent results depending on the indication and study population. Marr et al compared VOR plus CAS vs VOR alone in 47 patients with hematologic malignancy and hematopoietic cell transplant recipients with IA whose initial therapy with AmB failed. Improved 3-month survival was observed in the combination group as compared with monotherapy (hazard ratio, 0.42; 95% CI, .17–1.1; *P* = .048), independent of other variables associated with prognosis [64]. However, in a retrospective study spanning 12 years (1998–2010), VOR plus CAS did not achieve better outcomes than VOR alone, as primary or salvage therapy [65]. In solid organ transplant recipients, as compared with a historical cohort receiving L-AmB alone, VOR plus CAS for primary therapy of IA led to similar overall survival at day 90 (67.5% [combination] vs 51% [L-AmB monotherapy controls], *P* = .117) [66].

To date, 1 RCT has evaluated echinocandin-triazole combination for IA. This multicenter, multinational study randomized patients with hematologic malignancy or hematopoietic cell transplant recipients to VOR plus anidulafungin vs VOR alone for primary therapy of proven or probable IA [67]. Among 277 patients in the modified intention-to-treat analysis, mortality rates at 6 weeks were 19.3% for combination therapy and 27.5% for monotherapy (*P* = .087). Although the difference in mortality was not statistically significant, the number needed to treat of 13 to prevent death may be considered clinically significant. In a post hoc subgroup analysis of probable IA cases (n = 218), 6-week mortality was lower in the combination arm (15.7% vs 27.3% for monotherapy, *P* = .037). A limitation of this trial is that statistical power was lower than expected due to higher-than-expected mortality in both treatment arms.

Taken together, single studies have failed to decisively establish a benefit to CAF for IA. Given the time and resources involved, it is unlikely that another RCT of echinocandin-triazole combination therapy will be conducted. However, compelling preclinical data and observational clinical studies suggest potential benefit, and this combination may be considered for severe disease, especially in patients with hematologic malignancy [14].

#### Mucormycosis

Twenty-one articles, 6 retrospective studies and 15 case reports/series, were included in the systematic review. A 2008 retrospective study by Reed et al was one of the first to suggest a benefit to polyene-echinocandin combination therapy for primary treatment of rhino-orbital-cerebral mucormycosis. Among 41 patients with rhino-orbital-cerebral mucormycosis (83% diabetic) between 1994 and 2006 at 2 centers, 100% (7 of 7) receiving lipid-complex AmB or L-AmB plus CAS were alive and not in hospital care 30 days after hospital discharge vs 45% (15/34) receiving dAmB or a lipid formulation alone (*P* = .02) [68]. In multivariate analysis, only receipt of combination therapy was significantly associated with improved outcome (odds ratio, 10.9; *P* = .02).

Kyvernitakis et al performed the largest analysis to date in a retrospective study including 106 patients with hematologic malignancy and hematopoietic cell transplant recipients with mucormycosis at a single US cancer center between 1994 and 2014 [69]. For initial therapy, 44% of patients received monotherapy (L-AmB, 87%; POS, 13%) and 56% received CAF (L-AmB + echinocandin, 46%; L-AmB + POS, 27%; triple therapy, 27%), highlighting the frequency with which CAF is employed in clinical practice. Although this was a nonrandomized study, a well-done propensity score–adjusted analysis found a similar 6-week survival between patients receiving initial monotherapy and combination therapy (56% vs 60%, *P* = .71).

An important feature of the aforementioned studies is the relatively long study period, spanning >10 years, which may introduce biases pertaining to changes in fungal diagnostics, supportive care practices, and availability of specific antifungal agents and formulations. An additional limitation to observational studies of mucormycosis treatment is the use of different dosages and formulations of antifungal agents, which may affect efficacy and toxicity. It is particularly challenging and likely impractical to conduct RCTs for rare IFDs such as mucormycosis. Novel clinical trial designs merit careful consideration—for example, a prospective single-arm trial of CAF with an external contemporaneous control group from an established registry of IFD cases [70].

## ANIMAL STUDIES OF CAF

In the absence of large-scale RCTs, animal models of IFDs have several strengths. They allow for pharmacokinetics of the administered drugs, tissue burden, and rate of clearance to be assessed. Researchers can integrate host factors and study a range of doses and dose combinations. Finally, resistant species may be studied, whereas enrolling patients with rare and highly resistant fungal pathogens into clinical trials is quite challenging. With the implementation of ARRIVE (Animal Research: Reporting In Vivo Experiments) and ARRIVE 2.0 guidelines on the design, conduct, and reporting of animal studies, it is reasonable to conclude that animal studies with IFD and CAF will be able to bridge the quality gap with RCTs [71–73].

### *Candida* spp

Studies of CAF in experimental models of infections due to *Candida* spp have yielded mixed results depending on the species, drugs, and methodology. For example, the combination of FLC plus CAS for murine candidemia (*C albicans*) yielded no change in tissue fungal burden as compared with FLC monotherapy, whereas POS plus CAS led to improved survival vs either drug alone [74, 75].

An in vivo study by Louie et al further illustrates the complexity of antifungal interactions [76, 77]. In a rabbit model of *C albicans* endocarditis and pyelonephritis, AmB monotherapy and sequential treatment with AmB alone, followed by AmB plus FLC, rapidly sterilized kidneys and cardiac vegetations. In contrast, simultaneous AmB plus FLC, FLC monotherapy, and FLC, followed by AmB, were all slower to clear fungi from infected tissues [77].

Polyene-echinocandin combination is the most frequently studied regimen in the experimental models of IC, particularly due to *C glabrata*. When compared with monotherapy, AmB plus CAS or L-AmB plus CAS or micafungin led to reduced tissue fungal burden in a murine model of systemic infection due to azole-resistant *C albicans* and *C glabrata* and in immunosuppressed mice with *C glabrata* infection [78–80].

### *Cryptococcus* spp

In vivo studies of CAF for cryptococcosis laid the groundwork for subsequent RCTs in humans. In particular, AmB, FLC, and 5-FC, in different dosages and combinations, have been extensively studied in animal models of disseminated disease and meningitis. Schwarz et al demonstrated that AmB plus 5-FC, as compared with monotherapy, led to improved survival and reduced brain tissue fungal burden in murine disseminated cryptococcosis due to 5-FC–susceptible or 5-FC–resistant *C neoformans* [81]. Other studies have found that FLC and AmB, whether given sequentially or combined, led to greater antifungal activity than AmB alone [82, 83]. The role of high-dose FLC, with or without 5-FC, was further established in murine cryptococcal meningitis [84].

Other triazoles have received lesser attention, as monotherapy or combination therapy, in experimental models of cryptococcosis [85].

### *Aspergillus* spp

Preclinical studies of azole-echinocandin combinations for IA have garnered significant interest given the favorable safety profile of the latter group. The combinations of POS plus anidulafungin and ISA plus micafungin led to improved outcomes in neutropenic models of IA [86, 87]. This synergistic interaction between the echinocandin and the triazole is likely due to simultaneous inhibition of biosynthesis of 1,3-β-D-glucan in the fungal cell wall and ergosterol in the cell membrane. However, poorer outcomes observed with VOR plus high-dose anidulafungin suggest that azole-echinocandin interactions may be concentration dependent [88].

Experimental models of IA treated with azole-polyene combinations have primarily demonstrated antagonism except for central nervous system disease [88–91]. Again, consideration of the drug mechanism of action may explain these observations. Antifungal azoles deplete the fungal cell membrane of ergosterol and thereby diminish the principal biochemical target of AmB.

Polyene-echinocandin combinations did not show any benefit for experimental central nervous system aspergillosis or for invasive pulmonary aspergillosis in immunosuppressed murine models due to chronic granulomatous disease or glucocorticoids [91–93].

### Mucorales

Azole-polyene combinations for treatment of mucormycosis are frequently used in clinical practice, although preclinical data do not clearly establish a benefit. In neutropenic mice with pulmonary mucormycosis due to *Rhizopus delemar* or *Mucor circinelloides*, L-AmB plus ISA improved survival and reduced tissue fungal burden (lung and brain) as compared with either drug alone [94]. However, in murine disseminated mucormycosis due to *Rhizopus oryzae*, L-AmB plus POS did not improve outcomes as compared with L-AmB monotherapy [95, 96].

Echinocandins do not have in vitro activity against Mucorales in standard susceptibility tests; however, some agents of mucormycosis, including *R oryzae*, express (1→3)-β-D-glucan synthase, which is the target enzyme for echinocandins [97]. Experimental studies of murine mucormycosis due to *R oryzae* have found improved outcomes when combining lipid formulations of AmB plus echinocandins as compared with either agent alone [98, 99]. Yet, an Eagle effect is evident in that only lower doses of CAS and micafungin synergistically improve survival and reduce tissue fungal burden [89, 90]. The benefit noted with the addition of echinocandins may be in part due to the class’s immunomodulatory effect on the activity of phagocytic cells [100]. Nevertheless, no evidence of synergy or antagonism was noted for the combination of ISA and micafungin in neutropenic mice with pulmonary disease due to *R delemar* [101].

## LABORATORY METHODS

There are no standard methods from the CLSI (Clinical and Laboratory Standards Institute) or EUCAST (European Committee on Antimicrobial Susceptibility Testing) for in vitro testing of antifungal agents in combinations [102]. Thus, the utility of in vitro combination testing to inform clinical management of IFDs is uncertain yet suggested in several case reports [103–106]. Our literature analysis revealed numerous publications on laboratory testing of antifungal drugs in combinations against yeasts and molds (Supplementary Figure 1). Only a small selection of published studies included details consistent with the inoculum, inclusion of quality control strains, end point reading, and interpretations of test results per CLSI or EUCAST methods for testing of molds and yeasts [39, 107–124]. Even publications describing combination testing with standard methods originated from single institutions, and the reproducibility of the described method at other institutions remains unknown. The development of a consensus method with standardized parameters is imperative for the meaningful testing of antifungal combinations in the clinical laboratory. Toward this end, one of us conducted a series of multilaboratory studies to define the parameters that would allow reproducible antifungal combination testing. We identified a *Candida krusei* quality control strain, inoculum size, and 100% inhibition end point as highly reproducible features for combination testing of *Candida* species and *Aspergillus fumigatus* among 6 laboratories [125–127]. The result interpretations were consistent among laboratories according to summation fractional inhibitory concentration indices (ΣFICI) [125, 128]. Recent reanalysis of the data revealed significant pharmacodynamic interactions, with a Loewe additivity-based FIC index range of 1 to 2 and with essential agreement with Bliss independence–based response surface analysis [128]. Thus, laboratories now have a reproducible method for evaluation of in vitro testing of antifungal agents in combinations. Further progress is imminent when multilaboratory studies are conducted with newer antifungal combinations and with other fungal pathogens. Similarly, there is an opportunity for device manufacturers to offer commercial combination panels for wider access to routine laboratory testing.

## FUTURE PROSPECTS AND THE PATH FORWARD

Our systematic review of the literature from 2003 to 2023 highlights the relative paucity of controlled prospective studies of CAF for treatment of invasive infections due to *Candida* spp, *Aspergillus* spp, and the Mucorales, in contrast to the success of CAF studies for cryptococcal meningitis in individuals with HIV. We propose several priorities to advance CAF research efforts:

- Multilaboratory studies to investigate antifungal combinations for non-*Candida* fungal pathogens with facile reproducible methods [128]
- Development of sensitive and specific biomarkers to serve as surrogate efficacy end points
- Prioritization of CAF regimens for clinical trials based on animal models demonstrating improved survival and observational data supporting efficacy and safety
- Acceptance and implementation of novel trial designs for rare IFDs, such as target trial emulation [129]

Table 2 summarizes ongoing clinical trials of CAF, including those incorporating investigational and nonsystemic (eg, aerosolized) antifungal agents. Promising drugs in clinical phases of development with novel mechanisms of action include fosmanogepix, olorofim, and ibrexafungerp [130]. These agents offer hope to expand our antifungal armamentarium, particularly against resistant yeasts and molds, yet they will face the same environmental and host pressures for emergence of resistance over time. As such, drug development research should investigate these compounds in combinations and even look toward an antifungal “polypill” that might improve patient adherence and lessen health care costs [23, 131].

**Table 2.** Ongoing Clinical Trials of Combination Antifungal Therapy for Invasive Fungal Diseases

ClinicalTrials.gov ID	Phase	Sponsor	Disease	Treatment Arms	Primary End Point	Status
NCT03672292: Scynergia Trial	2	Scynexis, Inc; USA	Invasive pulmonary aspergillosis	1: Ibrexafungerp + voriconazole 2: Voriconazole + placebo	Adverse events, death through study completion	Completed, no results posted
NCT05238116: OPERA-T Trial	3	Pulmocide Ltd; UK	Refractory invasive pulmonary aspergillosis	1: Opelconazole (inh) + systemic antifungal therapy 2: Placebo (inh) + systemic antifungal therapy	Complete or partial overall response up to 12 wk	Recruiting
NCT05541107: EnACT3 Trial	3	Matinas BioPharma Nanotechnologies, Inc; USA	Cryptococcal meningitis	1: cAmB + flucytosine induction, followed by cAmB + fluconazole consolidation 2: cAmB + 5 flucytosine induction, followed by SOC consolidation 3: SOC induction, followed by SOC consolidation	All-cause mortality at 2 wk	Not yet recruiting
NCT04502381	2	Postgraduate Institute of Medical Education and Research; India	Pulmonary mucormycosis	1: Inh deoxycholate AmB + IV liposomal AmB 2: IV liposomal AmB	Overall response (clinical and radiologic) at 6 wk	Completed, no results posted

Abbreviations: AmB, amphotericin B; cAmB, encochleated amphotericin B; Inh, inhaled; IV, intravenous; SOC, standard of care.

While our review focused on a combinatorial strategy involving antifungal drugs, a particularly novel and emerging area of research is the role of cancer immunotherapies as adjunctive therapy for IFDs. Evidence that fungi induce activation of checkpoint pathways has led to preclinical data demonstrating that blockade of the programmed cell death protein 1 and cytotoxic T-lymphocyte antigen 4 pathways augments antifungal immunity and improves outcomes [132]. Thus far, clinical data are limited to case reports for invasive mold diseases [133, 134]. Besides providing support toward future interventional trials, such investigations of immunomodulatory therapies to combat IFDs highlight the need to understand the role of host immune responses in the outcomes of IFDs when designing and interpreting experimental and clinical studies of CAF.

## CONCLUSION

Our systematic review of the literature highlights the paucity of rigorous clinical investigations of CAF for IC and mucormycosis, whereas multiple RCTs establish the superiority of polyene-5-FC CAF for cryptococcosis and 1 large RCT of azole-echinocandin CAF for IA suggests a benefit in patients with hematologic malignancy and probable IA. Given the challenges to conducting large prospective RCTs for most IFDs, it is reasonable to prioritize future trials of CAF based on animal models demonstrating improved survival and observational clinical data supporting efficacy and safety. Recent multilaboratory investigations demonstrated the feasibility of a standardized method of in vitro testing of antifungal combinations for *Candida* spp that should be further investigated with other pathogen and drug combinations.

## Supplementary Material

ofae646_Supplementary_Data

## Supplementary Data

Supplementary materials are available at *Open Forum Infectious Diseases* online. Consisting of data provided by the authors to benefit the reader, the posted materials are not copyedited and are the sole responsibility of the authors, so questions or comments should be addressed to the corresponding author.

## Notes

***Author contributions.*** S. E. J. and V. C.: conceptualization, systematic literature review, writing of original draft, and review and editing.

***Patient consent statement.*** Not applicable.
