# Supplementary material for: CAF to the Rescue! Potential and Challenges of Combination Antifungal Therapy for Reducing Morbidity and Mortality in Hospitalized Patients With Serious Fungal Infections
Source: Open Forum Infect Dis. 2024 Oct 30;11(11):ofae646. doi: 10.1093/ofid/ofae646 (PMC11561589; doi:10.1093/ofid/ofae646)
Supplement: ofae646_Supplementary_Data [file ofae646_supplementary_data.pdf]

Appendix

Keywords used for the PubMed Search strategy:

#1

(("candida"[Title/Abstract] OR "candidiasis"[Title/Abstract]) AND "combination therapy"[Title/Abstract] AND 2003/01/01:3000/12/31[Date - Publication]) OR (("candida"[Title/Abstract] OR "candidiasis"[Title/Abstract]) AND "combination antifungal therapy"[Title/Abstract] AND 2003/01/01:3000/12/31[Date - Publication])

#2

(("cryptococcus"[Title/Abstract] OR "cryptococcal"[Title/Abstract] OR "cryptococcosis"[Title/Abstract]) AND "combination therapy"[Title/Abstract] AND 2003/01/01:3000/12/31[Date - Publication]) OR (("cryptococcus"[Title/Abstract] OR "cryptococcal"[Title/Abstract] OR "cryptococcosis"[Title/Abstract]) AND "combination antifungal therapy"[Title/Abstract] AND 2003/01/01:3000/12/31[Date - Publication])

#3

(("aspergillus"[Title/Abstract] OR "aspergillosis"[Title/Abstract]) AND "combination therapy"[Title/Abstract] AND 2003/01/01:3000/12/31[Date - Publication]) OR (("aspergillus"[Title/Abstract] OR "aspergillosis"[Title/Abstract]) AND "combination antifungal therapy"[Title/Abstract] AND 2003/01/01:3000/12/31[Date - Publication])

#4

(("mucormycosis"[Title/Abstract] OR "mucorales"[Title/Abstract] OR "zygomycetes"[Title/Abstract] OR "zygomycosis"[Title/Abstract]) AND "combination therapy"[Title/Abstract] AND 2003/01/01:3000/12/31[Date - Publication]) OR (("mucormycosis"[Title/Abstract] OR "mucorales"[Title/Abstract] OR "zygomycetes"[Title/Abstract] OR "zygomycosis"[Title/Abstract]) AND "combination antifungal therapy"[Title/Abstract] AND 2003/01/01:3000/12/31[Date - Publication])

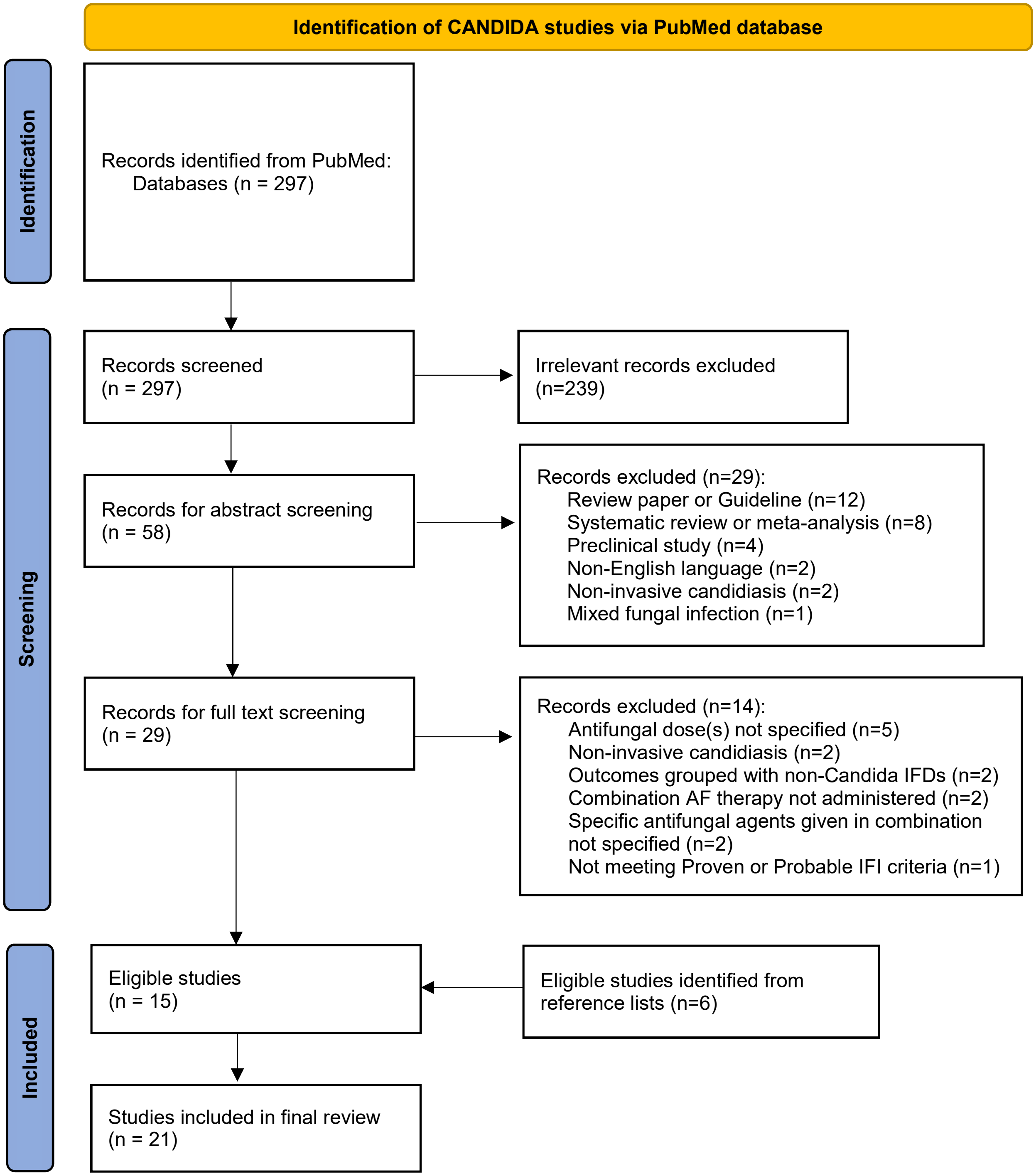
Identification of CANDIDA studies via PubMed database
Identification
Records identified from PubMed:
Databases (n = 297)
Screening
Records screened
(n = 297)
Irrelevant records excluded
(n=239)
Records for abstract screening
(n = 58)
Records excluded (n=29):
Review paper or Guideline (n=12)
Systematic review or meta-analysis (n=8)
Preclinical study (n=4)
Non-English language (n=2)
Non-invasive candidiasis (n=2)
Mixed fungal infection (n=1)
Records for full text screening
(n = 29)
Records excluded (n=14):
Antifungal dose(s) not specified (n=5)
Non-invasive candidiasis (n=2)
Outcomes grouped with non-Candida IFDs (n=2)
Combination AF therapy not administered (n=2)
Specific antifungal agents given in combination
not specified (n=2)
Not meeting Proven or Probable IFI criteria (n=1)
Included
Eligible studies
(n = 15)
Eligible studies identified from
reference lists (n=6)
Studies included in final review
(n = 21)

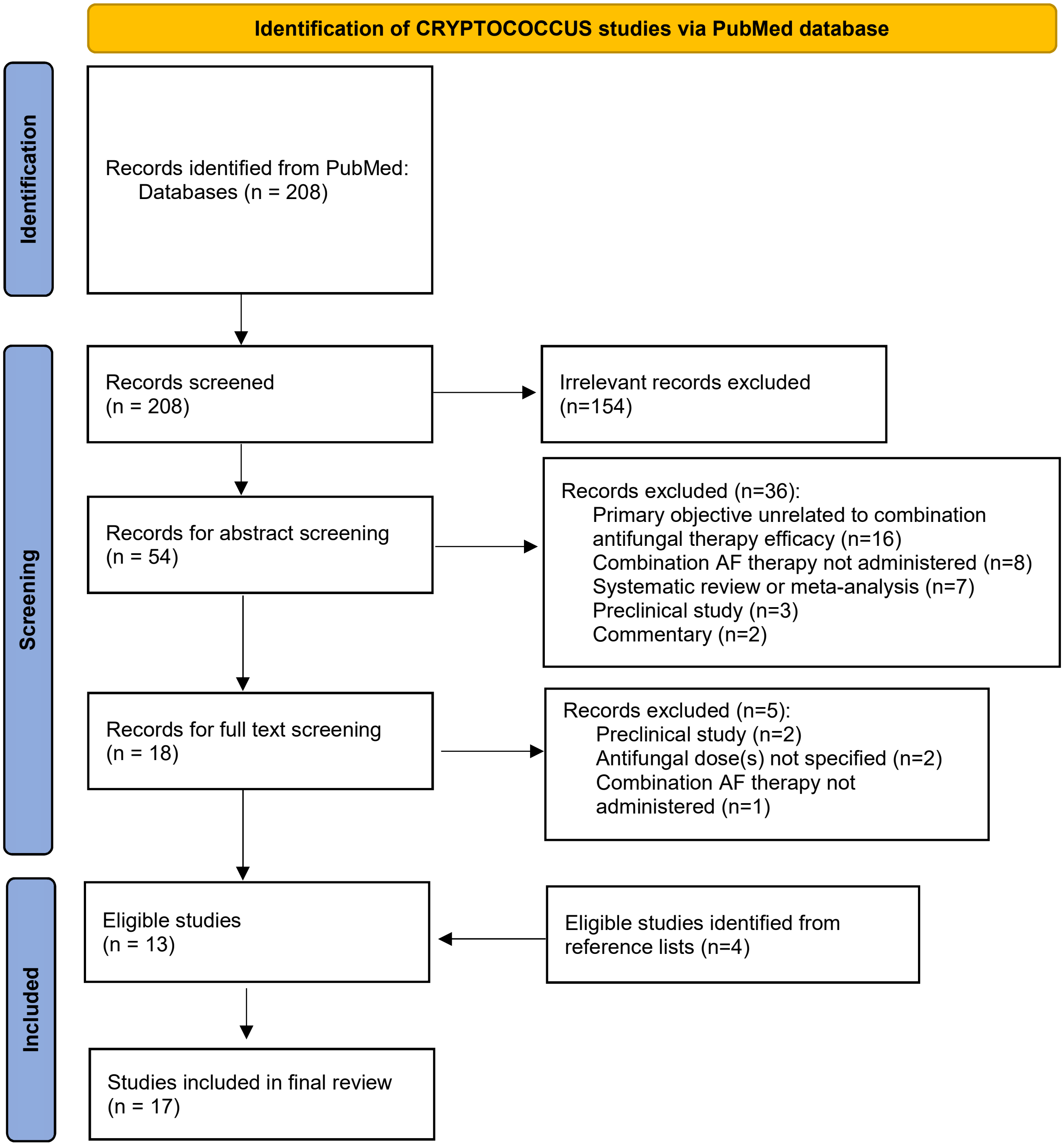
Identification of CRYPTOCOCCUS studies via PubMed database
Identification
Records identified from PubMed:
Databases (n = 208)
Screening
Records screened
(n = 208)
Irrelevant records excluded
(n=154)
Records for abstract screening
(n = 54)
Records excluded (n=36):
Primary objective unrelated to combination antifungal therapy efficacy (n=16)
Combination AF therapy not administered (n=8)
Systematic review or meta-analysis (n=7)
Preclinical study (n=3)
Commentary (n=2)
Records for full text screening
(n = 18)
Records excluded (n=5):
Preclinical study (n=2)
Antifungal dose(s) not specified (n=2)
Combination AF therapy not administered (n=1)
Included
Eligible studies
(n = 13)
Eligible studies identified from reference lists (n=4)
Studies included in final review
(n = 17)

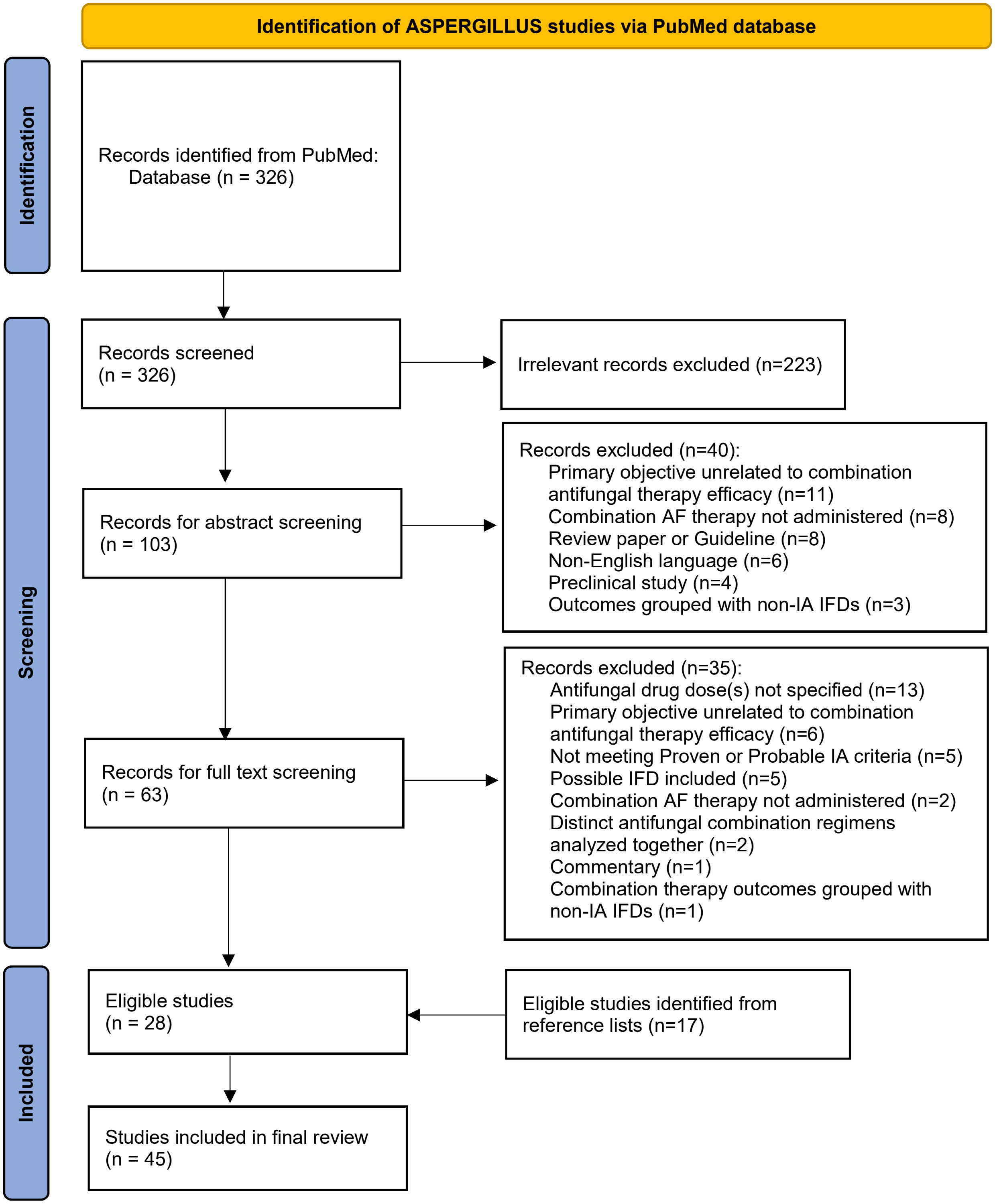
Identification of ASPERGILLUS studies via PubMed database
Identification
Records identified from PubMed: Database (n = 326)
Screening
Records screened (n = 326)
Irrelevant records excluded (n=223)
Records for abstract screening (n = 103)
Records excluded (n=40):
Primary objective unrelated to combination antifungal therapy efficacy (n=11)
Combination AF therapy not administered (n=8)
Review paper or Guideline (n=8)
Non-English language (n=6)
Preclinical study (n=4)
Outcomes grouped with non-IA IFDs (n=3)
Records for full text screening (n = 63)
Records excluded (n=35):
Antifungal drug dose(s) not specified (n=13)
Primary objective unrelated to combination antifungal therapy efficacy (n=6)
Not meeting Proven or Probable IA criteria (n=5)
Possible IFD included (n=5)
Combination AF therapy not administered (n=2)
Distinct antifungal combination regimens analyzed together (n=2)
Commentary (n=1)
Combination therapy outcomes grouped with non-IA IFDs (n=1)
Included
Eligible studies (n = 28)
Eligible studies identified from reference lists (n=17)
Studies included in final review (n = 45)

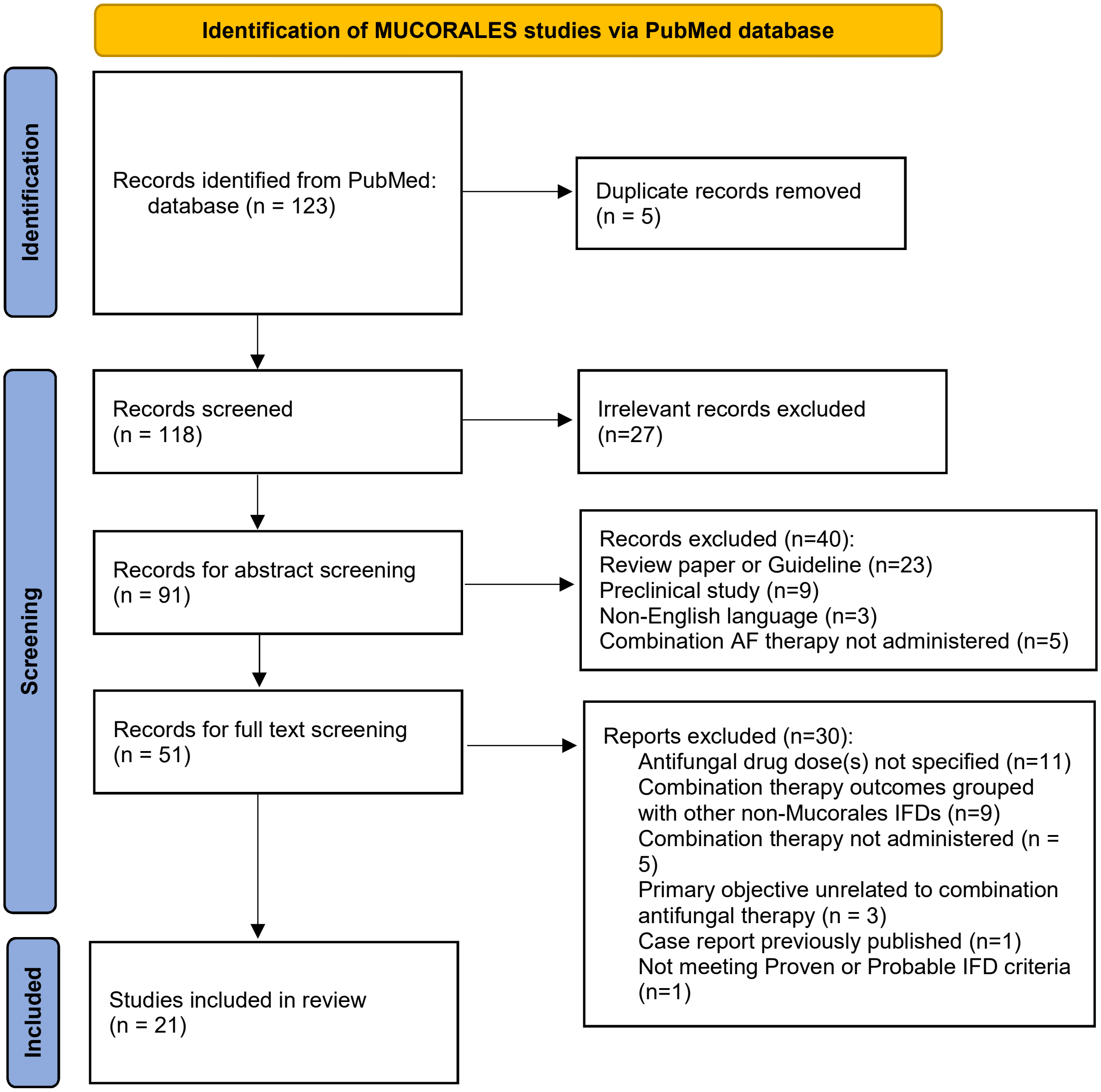
Identification of MUCORALES studies via PubMed database
Identification
Records identified from PubMed: database (n = 123)
Duplicate records removed (n = 5)
Screening
Records screened (n = 118)
Irrelevant records excluded (n=27)
Records for abstract screening (n = 91)
Records excluded (n=40):
Review paper or Guideline (n=23)
Preclinical study (n=9)
Non-English language (n=3)
Combination AF therapy not administered (n=5)
Records for full text screening (n = 51)
Reports excluded (n=30):
Antifungal drug dose(s) not specified (n=11)
Combination therapy outcomes grouped with other non-Mucorales IFDs (n=9)
Combination therapy not administered (n = 5)
Primary objective unrelated to combination antifungal therapy (n = 3)
Case report previously published (n=1)
Not meeting Proven or Probable IFD criteria (n=1)
Included
Studies included in review (n = 21)

Supplementary data

Table S1. Systematic review of publications evaluating combination antifungal therapy for invasive candidiasis, 2003-2023

1st author, year	Study design	Study period	Study population	N	Antifungal therapy†	Treatment indication	Results - Primary endpoint	Safety outcomes	Pediatric patients	Comments
Mikamo, 2003[1]	Case report	2002	29F solid tumor	1	FLC 400 mg/d + dAmB 0.5 mg/kg/d	Primary therapy, *C. glabrata* peritonitis	Resolved	None	N	
Rex, 2003[2]	Prospective, randomized, blinded, multicenter	1995-1999	Non-neutropenic hospitalized	219	FLC 800 mg/kg + PBO vs FLC 800 mg/d + dAmB 0.7 mg/kg/d	Initial therapy for candidemia	No difference in time-to-failure between groups, P = 0.08	Renal dysfunction in 3% (FLC + PBO) vs. 23% (FLC + dAmB), P < 0.001	Yes, >= 13 years-old	PBO or AmB component only for first 5-8 days of therapy; overall success rates 56% of FLC + PBO vs. 69% of FLC + dAMB (P = 0.043) while the bloodstream infection failed to clear in 17% and 6% of subjects, respectively (P = 0.02)
Pelletier, 2005[3]	Case report	NR	49F intestinal perforation	1	ABLC 5 mg/kg/d + 5-FC 2 g BID, followed by VOR + 5-FC	Salvage therapy, disseminated *C. krusei* including CNS	Resolved	NR	N	Progressive infection on CAS monotherapy for initial treatment
Sim, 2005[4]	Case report	2002	53M ALL	1	FLC 600 mg/d + CAS	Salvage therapy, *C. tropicalis* septic arthritis	Resolved	none	N	
Lopez-Ciudad, 2006[5]	Case report	NR	59F cirrhosis	1	VOR + CAS	Salvage therapy, *C. parapsilosis* mural endocarditis	Resolved	None	N	VOR added to CAS after 72 hours of initial therapy; surgical management deferred due to comorbidities

Olver, 2006[6]	Case report	NR	24M ALL	1	L-AmB 5 mg/kg/d + CAS	Salvage therapy, *C. krusei* bloodstream infection	Resolved	none	N	
Wong, 2008[7]	Retrospective case series	2000-2005	Continuous ambulatory peritoneal dialysis	13	dAmB 0.75-1 mg/kg/d + 5-FC 1 g/d	Primary therapy, *Candida* peritonitis	Microbiologic clearance in 10 (76.9%), 2 (15.4%) deaths related to peritonitis	Due to elevated LFTs, 5-FC stopped early in 2 patients, and both 5-FC and dAmB stopped early in 1 patient	N	Peritoneal catheter removed in 9 patients; outcomes also compared to historical control group of 14 patients (12 treated with antifungal monotherapy) that had higher peritonitis-related mortality (4/14, 28.5%)
Filippi, 2009[8]	Case report	NR	Neonate	1	FLC 6 mg/kg q 48 h + L-AmB 3 mg/kg/d + CAS 1 mg/kg/d	Salvage therapy, *C. albicans* bloodstream infection	Resolved	None	Y	
Bland, 2009[9]	Case report	2008	55F rheumatoid arthritis	1	MFG 100 mg/d + FLC 400 mg/d x 8 weeks, then FLC alone	Primary therapy, *C. albicans* prosthetic joint infection	Initial clinical and microbiological resolution, then clinical relapse on FLC monotherapy requiring hardware removal and reinitiation of combination therapy	None	N	
Turan, 2011[10]	Retrospective case series	2006-2009	Neonate	6	VOR 6 mg/kg q 8 h + L-AmB 5 mg/kg/d	Salvage therapy, *Candida* sepsis	Resolved	Elevated LFTs (n=4)	Y	Primary therapy included FLC alone, FLC + dAmB, or FLC + L-AmB

Al-Sweih, 2015[11]	Case report	NR	Neonate	1	L-AmB 8.5 mg/kg/d + CAS 2.5 mg/kg/d	Salvage therapy, *C. fermentati* bloodstream infection	Resolved	None	Y	
Reyes, 2015[12]	Case report	NR	36F ovarian cancer	1	AFG 100 mg/d + VOR 200 mg BID	Initial therapy, *C. parapsilosis* endocarditis	Resolved	None	N	Fluconazole suppression following 60 days x AFG + VOR
Mpakosi, 2016[13]	Case report	NR	Neonate	1	L-AmB 7 mg/kg/d + MFG 10 mg/kg/d	Salvage therapy, *C. pulcherrima* bloodstream infection	Resolved	none	Y	Checkerboard broth microdilution, synergy between AmB and MFG (FIC 0.375)
Al-Sweih, 2017[14]	Case report	NR	Neonate	1	L-AmB 8.5 mg/kg/d + CAS 2.5 mg/kg/d	Salvage therapy, *C. conglobata* bloodstream infection	Resolved	none	Y	
Tsakiri, 2018[15]	Case report	NR	Neonate	1	L-AmB 5 mg/kg/d + MFG 10 mg/kg/d + VOR 9 mg/kg q 12 h + intrathecal AmB 0.025 mg x 1 dose	Salvage therapy, *C. glabrata* meningitis with ventricular access device	Initial CSF sterilization, then relapse 34 days later that resolved after removal of hardware	none	Y	
Ahuja, 2019[16]	Case series	NR	Prosthetic valve endocarditis	2	Case 1: MFG 150 mg/d + FLC 400 mg/d x 12 weeks, then FLC 200 mg/d suppression;<br>Case 2: L-AmB 5 mg/kg/d + 5-FC 100 mg/kg/d + FLC 400 mg/d, then MFG 150 mg/d + 5-FC + FLC, then 5-FC + FLC suppression	Salvage therapy, *C. parapsilosis* PVE	Resolved	AKI on L-AmB	N	

Keuning, 2019[17]	Case report	2018	72F rheumatoid arthritis	1	VOR + MFG 200 mg/d x 4 weeks, then VOR 300 mg BID alone	Primary therapy, *C. parapsilosis* prosthetic joint infection	Resolved	Elevated LFTs attributed to MFG	N	Two-stage exchange arthroplasty with antifungal spacer (AmB) performed
Wee, 2021[18]	Case report	NR	Elderly F hemorrhagic stroke	1	L-AmB 5 mg/kg/d + 5-FC 100 mg/kg/d x 14 days, then FLC 12 mg/kg/d x 35 days	Primary therapy, *C. glabrata* meningitis	Resolved	None	N	
Jacobs and Jacobs, 2022[19]	Case report	2020	28F multivisceral transplant	1	VOR + MFG 150 mg/kg/d + ibrexafungerp 750 mg daily	Salvage therapy, *C. auris* peritonitis	Died	Leukopenia related to 5-FC	N	
Odysseos, 2022[20]	Case report	NR	52F liver transplant	1	L-AmB 6 mg/kg/d + ISA	Salvage therapy, *C. glabrata* bloodstream infection and bilomas	Resolved	None	N	
Sharma, 2023[21]	Case report	NR	55M rheumatic heart disease	1	CAS 150 mg/d + FLC 400 mg/d x 28 days, then FLC 400 mg/d	Primary therapy, *C. parapsilosis* prosthetic valve endocarditis	Died	None	N	Valve surgery deferred due to cachexia

5-FC, flucytosine; AFG, anidulafungin; AKI, acute kidney injury; ALL, acute lymphoblastic leukemia; CAS, caspofungin; CSF, cerebrospinal fluid; dAmB, deoxycholate amphotericin B; FLC, fluconazole; L-AmB, liposomal amphotericin B; LFTs, liver function tests; MFG, micafungin; NR, not reported; PBO, placebo; PVE, prosthetic valve endocarditis; SAE, serious adverse event

† Antifungal dosages unless otherwise specified: CAS 70 mg x 1 dose followed by 50 mg/d, VOR 6 mg/kg q 12 h x 2 doses followed by 4 mg/kg q 12 h, ISA 372 mg q 8 h x 6 doses followed by 372 mg/d

Table S2. Systematic review of publications evaluating combination antifungal therapy for cryptococcosis, 2003-2023

1st author, year	Study design	Study period	Study population	N	Antifungal therapy†	Treatment indication	Results - Primary endpoint	Safety outcomes	Pediatric patients	Comments
Brouwer, 2004[22]	Randomized trial	2002, Thailand	HIV/AIDS	64	dAmB 0.7 mg/kg/d alone vs. dAmB + 5-FC 100 mg/kg/d vs. dAmB + FLC 400 mg/d vs. triple therapy x 14 days, followed by FLC 400 mg/d	Induction therapy, CM	Higher EFA with dAmB + 5-FC vs. dAmB alone (P = 0.0006), dAmB + FLC (P = 0.02), or triple therapy (P = 0.02)	No study drug withdrawal due to side effects during first 2 weeks	N	No mortality difference across groups
Bicanic, 2008[23]	Randomized trial	2005-2006, South Africa	HIV/AIDS	64	dAmB 0.7 mg/kg/d + 5-FC 100 mg/kg/d vs. dAmB 1 mg/kg/d + 5-FC x 14 days, followed by FLC 400 mg/d	Induction therapy, CM	Higher EFA with dAmB 1 mg/kg/d + 5-FC than dAmB 0.7 mg/kg/d + 5-FC (P = 0.05)	No difference in renal impairment between groups	N	No mortality difference between groups
Milefchik, 2008[24]	Randomized trial	1991-1994	HIV/AIDS	89	FLC 800mg or 1200 mg or 1600 mg or 2000 mg/d x 10 weeks +/- 5-FC 100 mg/kg/d x 4 weeks	Induction therapy, CM	Alive with negative CSF culture by week 10: FLC 800 mg alone (11%) or plus 5-FC (75%), FLC 1200 mg alone (37%) or plus 5-FC (87%), FLC 1600 alone (62%) or plus 5-FC (62%), FLC 2000 mg alone (62%) or plus 5-FC (75%)	Elevated LFTs and granulocytopenia, respectively in 2% and 7% FLC alone and 7% and 18% combination groups	N	

Pappas, 2009[25]	Randomized trial, phase 2	2005-2007, U.S. and Thailand	HIV/AIDS	143	dAmB 0.7 mg/kg/d alone vs. dAmB 0.7 mg/kg/d + FLC 400 mg/d vs. dAmB 0.7 mg/kg/d + 800 mg/d x 14 days, followed by FLC alone at randomized dosage	Induction therapy, CM	No difference in rates of severe toxicity between groups; higher rate of successful outcome in dAmB + FLC 800 mg group (53.7%) vs. dAmB alone (41.3%) and dAmB + FLC 400 mg (27.1%)	Most common toxicities hypokalemia and hypomagnesemia	N	Primary efficacy endpoint = composite of CSF sterilization, stable neurological function, and survival at day 14
Nussbaum, 2010[26]	Randomized trial	2008, Malawi	HIV/AIDS	41	FLC 1200 mg/d alone vs. FLC + 5-FC 100 mg/kg/d x 14 days, followed by FLC 800 mg/d	Induction therapy, CM	Higher EFA in combination arm compared to monotherapy (P = <0.001)	More grade 3 or 4 neutropenia in combination arm	N	Fewer deaths in combination arm at 2 weeks (10% vs. 37%) and at 10 weeks (43% vs. 58%)
Jackson, 2012[27]	Randomized trial	2009-2010, Malawi	HIV/AIDS	40	FLC 1200 mg/d + dAmB 1 mg/kg/d vs. FLC + 5-FC 100 mg/kg/d + dAmB 1 mg/kg/d x 14 days, followed by FLC 800 mg/d	Induction therapy, CM	Higher EFA in triple combination arm compared to FLC + dAmB (P = 0.03)		N	No difference in mortality between groups
Loyse, 2012[28]	Randomized trial	2006-2008, South Africa	HIV/AIDS	80	dAmB 0.7-1 mg/kg/d + 5-FC 100 mg/kg/d vs. dAmB + FLC 800 mg/kd vs. dAmB + FLC 600 mg/d vs. dAmB + VOR 300 mg BID x 14 days, followed by FLC 400 mg/d	Induction therapy, CM	No difference in EFA among groups	No significant difference in grade 3 and 4 AEs among groups	N	

Muzoora, 2012[29]	Prospective, open-label, compared to historical cohort	2008-2009, Uganda	HIV/AIDS	90	dAmb 1 mg/kg/d x 5 days + FLC 1200 mg/d x 14 days (n=30) vs. historical cohort treated with FLC 800 mg/d or 1200 mg/d alone x 14 days (n=60)	Induction therapy, CM	More rapid EFA in combination group than FLC 800 mg or 1200 mg groups (P < 0.001 for both)	No grade 4-5 anemia or elevated Cr in combination group	N	Overall mortality 28% at 10 weeks in combination group
Day, 2013[30]	Randomized trial	2004-2010, Vietnam	HIV/AIDS	299	dAmB 1 mg/kg/d x 4 weeks vs. dAmB + 5-FC 100 mg/kg/d x 2 weeks vs. dAmB + FLC 400 mg BID x 2 weeks	Induction therapy, CM	Fewer deaths in dAmB + 5-FC arm compared to dAmB alone arm at day 14 (P = 0.08) and day 70 (P = 0.04); no difference in mortality between dAmB alone and dAmB + FLC arms; higher EFA in dAmB + 5-FC arm compared to both dAmB alone and dAmB + FLC arms	common in combination therapy groups compared to dAmB alone	N	First study to show a mortality benefit with combination therapy
Vaidhya, 2015[31]	Randomized trial	2012-2014, India	HIV/AIDS	84	dAmB 0.7-1 mg/kg/d alone vs. dAmB + FLC 400 mg/d x 14 days	Induction therapy, CM	No difference in rates of fever, headache, neck stiffness between groups at 14 days, but greater improvement in altered sensorium and focal neurologic deficits and lower rates of CSF culture positivity at day	NR	N	

							14 in combination arm vs. monotherapy.			
Molloy, 2018[32]	Randomized trial (ACTA)	2013-2016, Africa	HIV/AIDS	678	FLC 1200 mg/d + 5-FC 100 mg/kg/d x 2 weeks vs. dAmB 1 mg/kg/d + FLC 1200 mg/kg OR 5-FC 100 mg/kg/d x 1 week followed by FLC 1200 mg/d vs. dAmB 1 mg/kg/d + FLC OR 5-FC x 2 weeks	Induction therapy, CM	Similar mortality in the oral-regimen, 1-week dAmB, and 2-week dAmB groups: 18.2%, 21.9%, and 21.4%, respectively, at 2 weeks, and 35.1%, 36.2%, and 39.7%, respectively, at 10 weeks	Grade 4 anemia and grade 3-4 serum creatinine increase more common in dAmB groups compared to oral-regimen group	N	Lowest 10-week mortality in 1-week dAmB + 5-FC group (24.1%;95% CI, 16.2-32.1); more rapid CSF sterilization in dAmB + 5-FC groups compared to dAmB + FLC and to oral-regimen groups
Jandhyala, 2019[33]	Case report	NR	Critical illness	1	L-AmB 5 mg/kg/d + 5-FC 50 mg/kg/d x 2 weeks, followed by FLC 800 mg/d	Primary therapy, disseminated cryptococcosis (blood and lungs)	Survived	NR	N	

Jarvis, 2019[34]	Randomized trial	2014-2016, Botswana and Tanzania	HIV/AIDS	80	L-AmB 10 mg/g day 1 only vs. 10 mg/kg day 1 and 5 mg/kg day 3 vs. L-AmB 10 mg/kg day 1 and 5 mg/kg days 3 and 7 vs. L-AmB 3 mg/kg/d x 14 days (control arm). All groups received FLC 1200 mg/d x 14 days.	Induction therapy, CM	Non-inferior EFA in all short-course L-AmB arms compared to control arm.	Low rates of grade ≥ 3 clinical and laboratory AEs overall and no difference between treatment arms	N	No significant difference in 2-week and 10-week mortality between treatment arms.
Li, 2019[35]	Retrospective cohort study	2010-2017, China	Non-HIV, non-transplant	90	dAmB 0.57-0.98 mg/kg/d + 5-FC 55.75 +/- 14.30 mg/kg/d (n=43) vs. FLC 400-800 mg/d + 5-FC 57.64 +/- 13.34 mg/kg/d (n=47)	Induction therapy, CM	No difference in rate of CSF clearance at 12 weeks in dAmB (74.4%) vs. FLC (70.2%) groups, P = 0.814	Fewer AEs in FLC vs dAmB group.	N	At baseline, higher CSF cryptococci burden in FLC group. No difference in EFA, treatment response, or mortality (overall 3.3%) between groups.
Jarvis, 2022[36]	Randomized trial	2018-2012, Africa	HIV/AIDS	814	L-AmB 10 mg/kg x 1 dose + 5-FC 100 mg/kg/d and FLC 1200 mg/d vs. dAmB 1 mg/kg/d + 5-FC 100 mg/kg/d x days followed by FLC 1200 mg/d x 7 days (control arm)	Induction therapy, CM	10-week mortality non-inferior in L-AmB arm (24.8%) vs. control arm (28.7%).	Fewer life-threatening AEs in L-AmB arm (21.7%) vs. control arm (30.1%; P = 0.005) and less grade 3-4 anemia in L-AmB arm than control arm (P < 0.001)	N	No difference in EFA between treatment arms

Takazono, 2022[37]	Retrospective cohort study	2010-2017, Japan	Hospitalized patients without HIV (malignancy [29%], steroid usage [63.1%], collagen diseases [22.9%])	363	L-AmB alone (median dose 3.49 mg/kg/d, IQR 2.51-4.64), n=146 patients vs. L-AmB (median dose 3.75 mg/kg/d, IQR 2.78-4.71) + 5-FC, n=217 patients	Induction therapy, CM	No difference in mortality 14 days after diagnosis in L-AmB alone vs combination group (11% vs. 6%, respectively; HR 0.5775, 95% CI 0.28-1.21, P = 0.1)	NR	N	5-FC dose not specified
Zhao, 2022[38]	Retrospective cohort study	2013-2018, China	Persons without HIV (steroid usage [31.6%], diabetes mellitus [23.7%], liver cirrhosis [7.9%])	38	FLC 800 mg/d + 5-FC 78-100 mg/kg/d (n=27) vs. FLC 800 mg/d alone (n=11)	Initial therapy, CM	Successful response to initial therapy in 77.8% (combination arm) and 72.7% (monotherapy arm)†	No difference in rates of adverse drug reactions between groups; rate of leukopenia not reported	N	Median duration of initial therapy 41 days (IQR, 28.5–105.5)

5-FC, flucytosine; AE, adverse event; dAmB, deoxycholate amphotericin B; L-AmB, liposomal amphotericin B; CM, cryptococcal meningitis; CSF, cerebrospinal fluid; EFA, early fungicidal activity; FLC, fluconazole; VOR, voriconazole

† Successful response defined according to reference 39

Table S3. Systematic review of publications evaluating combination antifungal therapy for invasive aspergillosis, 2003-2023

1st author, year	Study design	Study period	Study population	N	Antifungal therapy	Treatment indication	Results - Primary endpoint	Safety outcomes	Pediatric patients	Comments
Elanjikal, 2003[40]	Case report	2001	2F ALL	1	L-AmB 5 mg/kg/d + CAS 2 mg/kg x 1 then 1.5 mg/kg/d	Salvage therapy, proven IPA	Survived	Hypokalemia with AmB	Y	

Lujber, 2003[41]	Case report	1999	25F immunocompetent	1	L-AmB 6-10 mg/kg/d + 5-FC 8 g/d	Salvage therapy, chronic invasive fungal rhinosinusitis with orbital and cerebral involvement	Survived	none	N	
Kontoyiannis, 2003[42]	Retrospective cohort study	2001	HM or HCT	48 (n=23 proven or probable IA)	L-AmB 5 mg/kg/d + CAS	Primary or salvage therapy, proven, probable or possible IA	Success in 2/6 (33%) initial therapy cases and 3/17 (18%) salvage cases	Mild to moderate renal insufficiency in 15%	N	
Damaj, 2004[43]	Case report	NR	57M AML	1	VOR + CAS	Salvage therapy, disseminated IA (lungs and CNS)	Survived	NR	N	IA initially progressed on dAmB monotherapy and dAmB + CAS combination
Marr, 2004[44]	Retrospective cohort study	1997-2001	HM or HCT	47	VOR (n=31) vs. VOR + CAS (n=16)	Salvage therapy proven or probable IA	Improved 3-month survival with VOR + CAS vs. VOR alone		N	All pts received AmB or L-AmB for primary therapy
Rosen-Wolff, 2004[45]	Case report	NR	13M CGD	1	LAmB 5 mg/kg/d + CAS 1 mg/kg/d	Primary therapy, proven IPA (*A. nidulans* and *A. fumigatus*)	Progressive infection requiring change in antifungal therapy to VOR monotherapy	Hypokalemia with AmB	Y	Infection resolution on VOR monotherapy
Castagnola, 2004[46]	Case series	NR	11M HCT	1	VOR + CAS 40 mg/m2/d loading, then 30 mg/m2/d	Salvage therapy, proven IA	Died	NR	Y	Death due to septic shock (Methicillin-resistant *Staphylococcus aureus*)
Elzi, 2005[47]	Case report	NR	49M kidney transplant	1	ABLC 5 mg/kg/d + CAS 50 mg/d + 5-FC 3.5 g/d	Primary therapy, disseminated IA	Died	Leukopenia related to 5-FC	N	
Schuster, 2005[48, 49]	Case report	NR	4M ALL	1	VOR + CAS 1 mg/kg/d	Salvage therapy, probable IPA	Survived	None	Y	

Kontoyiannis, 2005	Retrospective	1993-2003	HM or HCT	112	L-AmB 5 mg/kg/d (n=101), L-AmB + ITC (n=11)	Primary therapy proven or probable IA	No difference in response at end of primary therapy nor in 2-week or 4-week mortality between groups		N	Excluded subjects receiving ITC capsules
Pancham, 2005[50]	Case series	NR	14M HCT	1	L-AmB 5 mg/kg/d + CAS 50 mg/d	Salvage therapy, probable IPA (*A. fumigatus*)	Survived	None	Y	
Lehrnbecher, 2006[51]	Case report	NR	10M HCT	1	L-AmB 5 mg/kg/d + VOR	Salvage therapy, proven intestinal IA	Survived	Elevated LFTs requiring temporary switch from VOR to CAS	Y	Laparotomy and partial small bowel resection performed
Denning, 2006[52]	Prospective, open-label, non-comparative, multicenter	1998-2002	HCT, HM, SOT, HIV/AIDS, Others	225	MFG 75 mg daily with optional dose escalation alone (n=34) or in combination with other agents (n=191, primarily L-AMB)	Primary therapy (n=29) or salvage therapy (n=196) proven or probable IA	Primary therapy: Favorable response (CR+PR) at end of therapy in 29.4% (combination) vs 50% (MFG alone) Salvage therapy: Favorable response in 34.5% (combination) vs 40.9% (MFG alone)	MFG well-tolerated alone or in combination	Y	
Maertens, 2006[53]	Prospective, open-label, non-comparative, multicenter	2003-2004	HCT, HM, Corticosteroids	53	CAS + ITC (n=7) or VOR (n=30) or dAmB 0.75-1.5 mg/kg/d (n=5) or L-AmB ≥ 5 mg/kg/d (n=11)	Salvage therapy proven or probable IA	Favorable response at end of therapy in 55% (50% CAS + polyene group and 56.8% CAS + triazole group) Survival at Day 84 - 55%		N	Nine patients received combination therapy not including CAS as part of initial regimen

Singh, 2006[54]	Prospective, observational, multicenter	2003-2005	SOT	87	VOR + CAS (n=40) vs historical control group lipid formulation AmB alone 5-7.4 mg/kg/d (n=47)	Primary therapy proven or probable IA	No difference in overall survival Day 90 in combination (67.5%) vs control groups (51%)		N	Combination therapy associated with improved survival in subgroup with renal failure and in those with *A. fumigatus* infection; No correlation observed between in vitro antifungal interactions and clinical outcomes
Cesaro, 2007[55]	Prospective, multicenter surveillance study	2002-2005	Pediatric heme-onc or HCT	40	L-AmB 3-5 mg/kg/d plus CAS (n=18), VOR plus CAS (n=9), or both combinations sequentially (n=9)	Primary or salvage therapy proven or probable IA	Favorable response in 53%; 100-day survival probability 70%	Well-tolerated, no severe renal or liver impairment	Y	
Caillot, 2007[56]	Multicenter, randomized, open-label (Combistrat)	2004-2005	HM	30	L-AmB 3 mg/kg/d + CAS vs. L-AmB 10 mg/kg/d alone	Primary therapy proven or probable IA	Higher favorable response rate at end of therapy in combination (67%) vs. monotherapy group (27%) P = 0.028		Y	23% of patients in high-dose monotherapy group experienced 2-fold increase in serum Cr
Meyer, 2007[57]	Case report	2004	54M AML	1	VOR + dAmB 1.5 mg/kg/d	Salvage therapy, proven IPA	Progressive disease, requiring surgical resection	NR	N	Lower lobe resection required
Gubler, 2007[58]	Case report	NR	43M cirrhosis	1	VOR 50-200 mg BID + CAS	Primary therapy, disseminated IA (lungs and CNS, *A. fumigatus*)	Survived	NR	N	
Panda, 2008[59]	Case series	NR	Adult immunocompetent	6	dAmB 1-1.5 mg/kg/d (gradual dose escalation) + ITC 300 mg x 2 doses, then 200 mg BID	Primary therapy, invasive fungal sinusitis with orbital +/- intra-cranial extension	100% disease-free survival	NR	N	Surgical debridement in 1/6 patients

Raad, 2008[60]	Single center compassionate use trial	1994-2005	HM or HCT	143	POS 800 mg/d suspension (n=53) vs. historical controls L-AmB ≥ 7.5 mg/kg/d alone (n=52) or with CAS (n=38)	Salvage therapy proven or probable IA	Higher favorable response rate in POS group (40%) vs L-AMB alone or with CAS (8% and 11%, respectively)	Higher rate of nephrotoxicity and hepatotoxicity in L-AmB alone and combination group	N	Most patients (~65%) received L-AmB 3-5 mg/kg/d for primary therapy;
Saijo, 2008[61]	Case report	NR	56M alcoholic liver disease	1	MFG 300 mg/d + VOR 400 mg/d	Salvage therapy, probable IA (lung and CNS)	Survived	Liver dysfunction, reversed after VOR dose reduction	N	Initial therapy: MFG 150 mg/d
Matsuda, 2010[62]	Case report	2005	62M interstitial pneumonia on systemic immunosuppression	1	VOR 300 mg BID + MFG 150 mg/d	Salvage therapy, proven *Aspergillus* empyema	Clinical, radiographic, and mycological resolution	Elevated LFTs requiring VOR dose reduction	N	
Yamada, 2010[63]	Case report	2007	45M AML	1	L-AmB 200 m/d + MFG 300 mg/d, followed by VOR 400 mg/d + MFG	Salvage therapy, *Aspergillus* liver abscess	Clinical and radiographic resolution	Elevated serum creatinine attributed to L-AmB	N	
Aoki, 2011[64]	Case report	2007-2008	49F AML, 48F AML	1	Case 1: VOR + MFG 150 mg/d; Case 2: VOR 400 mg/d + MFG 150 mg/d	Salvage therapy, probable IPA	Survived	None	N	
Beiras-Fernandez, 2011[65]	Case report	NR	45M heart transplant	1	VOR 400 mg/d + MFG 100 mg/d	Primary therapy, IPA	Clinical and radiographic resolution	None	N	
Kerl, 2011[66]	Case report	NR	5M HCT	1	VOR 10 mg/kg BID + CAS 50 mg/d	Primary therapy, cutaneous IA	Resolved	Elevated liver transaminases and facial rash possibly related to VOR	Y	Surgical debridement performed

Lazaro, 2011[67]	Case report	2009	40M lung transplant	1	VOR 400 mg BID x 2 doses, then 300 mg BID + AFG	Primary therapy, *Aspergillus fumigatus* infectious endocarditis	Died	NR	N	
Dupouey, 2012[68]	Case series	NR	Aortic valve surgery	3	Case 1: VOR + CAS, Case 2: dAmB 70 mg/d + 5-FC 10 g/d, Case 3: CAS + L-AmB 5 mg/kg/d + VOR	Primary therapy, *A. flavus* infectious endocarditis	Case 1: survived, Cases 2 and 3: died	NR	N	
Doring, 2012[69]	Case report	2006	18M ALL and HCT x 3	1	L-AmB 3 mg/kg/d + CAS	Primary therapy, probable IPA	Clinical and radiographic improvement, then worsening after switch to VOR	Elevated blood urea nitrogen possibly related to L-AmB	N	
Rojas, 2012[70]	Retrospective, multicenter, observational study	2005-2010	HM or HCT	61 (n=49 with IA)	L-AmB 3 mg/kg/d + CAS (n=18), L-AmB 3 mg/kg/d + VOR or POS 400 mg q 12 h (n=14), or VOR + EC (n=17)	Initial therapy, proven or probable IA	Favorable response and 12-week survival, respectively, in 13/18 (72%) L-AmB + CAS group, 8/14 (57%)L-AmB + VOR or POS group, 11/17 (65%) VOR + EC group	Mild elevated LFTs (VOR) and mild increase serum creatinine (L-AmB)	Y	Pediatric VOR dosing 7 mg/kg q12 h
Pillai, 2013[71]	Case report	NR	47M pacemaker surgery	1	L-AmB 5 mg/kg/d + VOR 200 BID	Primary therapy, pacemaker lead *Aspergillus* endocarditis	Survived	None	N	
Demir, 2015[72]	Case report	NR	36M HCT	1	L-AmB 3 mg/kg/d + CAS	Salvage therapy, disseminated IA (lungs, heart, skin, CNS)	Died	NR	N	

Marr, 2015[73]	Randomized, double-blind, placebo-controlled, multicenter	2008-2011	HM or HCT	277	VOR alone (n=142) or with AFG (n=135)	Primary therapy proven or probable IA	No difference in 6-week all-cause mortality between groups: 19.3% (VOR + AFG) vs. 27.5% (VOR) p=0.087	Similar rates of TEAEs except more hepatobiliary disorders in combination group (12.7%) than monotherapy group (8.4%)	N	Post-hoc subgroup analysis of probable IA cases (n=218) found lower 6-week mortality in combination (15.7%) vs monotherapy (27.3%) groups; p=0.037 Therapeutic drug monitoring not performed
Raad, 2015[74]	Retrospective, single-center	1998-2010	HM	138	CAS alone (n=32), VOR alone (n=62) or with CAS (n=68)	Primary or salvage therapy proven or probable IA	No difference in favorable response rates among the 3 groups for primary or salvage therapy No difference in IA-associated mortality between VOR monotherapy and VOR + CAS groups for primary or salvage therapy	Primary therapy: no difference in adverse events between groups Salvage therapy: More adverse events in combination group (37%) vs CAS alone (6%)	Y	
Rofaiel, 2016[75]	Case report	NR	64F acute leukemia	1	L-AmB 5 mg/kg/d + 5-FC 25 mg/kg q 6 h	Primary therapy, *A. fumigatus* native valve endocarditis	Died	NR	N	
Nicolé, 2017[76]	Case report	2015	70M cryoglobulinemic vasculitis on corticosteroids	1	VOR 200 mg q 12 h + CAS	Salvage therapy, *Aspergillus* thyroiditis	Persistent thyroid abscesses requiring total thyroidectomy	Liver toxicity on VOR	N	
Chen, 2017[77]	Case report	NR	16M solid tumor	1	VOR 11 mg/kg q 12 h + CAS 100 mg/d	Salvage therapy, CNS aspergillosis	Resolved	Elevated liver transaminases	Y	

Lee, 2019[78]	Subgroup analysis from RCT (ref Marr)	2008-2011	HM or HCT (Korean patients only)	40	VOR alone (n=17) or with AFG (n=23)	Primary therapy proven or probable IA	Week 6 all-cause mortality numerically lower in combination arm (17.6%) than monotherapy arm (41.2%), a more marked reduction in mortality as compared to non-Koreans	No difference in TEAEs between groups	N	
Calvo-Cano, 2020[79]	Case report		56M kidney transplant	1	L-AmB 8 mg/kg/d + 5-FC 3 g 3x/d + inhaled AmB	Salvage therapy, azole-resistant disseminated IA (*A. parafelis*, lungs and CNS)	Progressive disease requiring neurosurgery (lobectomy)		N	Following neurosurgery, switched to MFG 350 3x/week + POS DR 300 mg/d maintenance therapy
Furudate, 2020[80]	Case report	NR	Preterm infant	1	VOR 4.5-9 mg/kg q 12 h + L-AmB 5 mg/kg/d	Salvage therapy, IPA and *Aspergillus* ventriculitis	Resolved	Elevated LFTs requiring temporary discontinuation of VOR	Y	Endoscopic intraventricular lavage also performed
Lennard, 2020[81]	Case report	2016	62M aortic valve replacement	1	L-AmB 5 mg/kg/d + VOR	Primary therapy, *Aspergillus fumigatus* prosthetic valve endocarditis	Resolved	NR	N	Aortic root debridement and aortic valve replacement performed
Mendoza, 2020[82]	Case report	NR	56F HCT	1	L-AmB 5 mg/kg/d + MFG 150 mg/d + TRB 500 mg q 12 h	Salvage therapy, disseminated IA (*A. calidoustus*)	Clinical and mycological response (downtrending serum GM)	AKI requiring switch from L-AmB to ISA	N	In vitro synergy testing performed but methodology not specified: synergy between ISA and TRB (MICs: 0.5 + 0.03 µg/mL, respectively) but no synergy (indifferent) for MFG + ISA combination (MICs: ≤ 0.06 + 2 µg/mL, respectively)

Camargo, 2022[83]	Case report	NR	40M HCT and GVHD	1	L-AmB 5 mg/kg/d + MFG 100-150 mg/d + ISA + TRB 500 mg BID + Fosmanogepix 1 g q 12 h x 2 doses then 600 mg/d	Primary therapy, disseminated IA (*A. calidoustus*)	Resolved	NR	N	Continued on fosmanogepix 400 mg BID alone as of last follow-up 324 days after IA diagnosis. In vitro synergy testing performed but methodology not specified: synergy between AmB + MFG (≤ 0.03), AmB + POS (≤ 0.03), AmB + VOR (≤ 0.03), AmB + TRB (≤ 0.015), AmB + ISA (≤ 0.03), ISA + TRB (≤ 0.03).
Nakaya, 2023[84]	Case report	NR	29F HCT and GVHD	1	VOR 300-600 mg/d + CAS 70 mg/d --> progressive disease --> ITC 200 mg/d + MFG 150 mg/d -> progressive disease --> POS 200-600 mg/d + L-AmB 5 mg/kg/d	Salvage therapy, COVID-19 associated pulmonary aspergillosis (*A. fumigatus*)	Resolved	Hypokalemia with AmB	N	

5-FC, flucytosine; ABLC, lipid complex amphotericin B; AKI, acute kidney injury; ALL, acute lymphoblastic leukemia; CAS, caspofungin; CGD, chronic granulomatous disease; CNS, central nervous system; dAmB, deoxycholate amphotericin B; GM, galactomannan; GVHD, graft-versus-host disease; HCT, hematopoietic cell transplantation; HM, hematologic malignancy; ITC, itraconazole; IPA, invasive pulmonary aspergillosis; L-AmB, liposomal amphotericin B; LFTs, liver function tests; MFG, micafungin; NR, not reported; PVE, prosthetic valve endocarditis; SAE, serious adverse event; TRB, terbinafine

† Antifungal dosages unless otherwise specified: AFG 200 mg x 1 dose followed by 100 mg/d, CAS 70 mg x 1 dose followed by 50 mg/d, ISA 372 mg q 8 h x 6 doses followed by 372 mg/d, ITC 200 mg BID x 2 days followed by 200 mg/d, VOR 6 mg/kg q 12 h x 2 doses followed by 4 mg/kg q 12 h or 200 mg q 12 h

Table S4. Systematic review of publications evaluating combination antifungal therapy for mucormycosis, 2003-2023

1st author, year	Study design	Study period	Study population	N	Antifungal therapy†	Treatment indication	Results - Primary endpoint	Safety outcomes	Pediatric patients	Comments
Rickerts, 2006[85]	Case report	2005	46F AML	1	L-AmB 5 mg/kg/d + POS 400 mg BID	Salvage, disseminated IM *(Rhizomucor* spp.)	Survived	NR	N	
Reed, 2008[86]	Retrospective cohort study, two centers	1994-2006	ROCM (83% diabetic, 34% cancer, 12% neutropenia, 10% transplant)	41	dAmB 1 mg/kg/d (n=15), ABLC 5 mg/kg/d (n=17), L-AmB 5 mg/kg/d (n=2); CAS + ABLC (n=5), CAS + L-AmB (n=2)	Primary therapy proven IM	Alive and not in hospital care 30 days after hospital discharge: 100% combination therapy vs. 45% monotherapy, P = 0.02	Doubling of serum Cr in 56% during therapy with no difference between polyene agent	Y	Among patients with CNS disease, primary endpoint met in 4/4 (100%) combination group vs. 25% monotherapy group
Lebeau, 2010[87]	Case report	NR	46F AML	1	L-AmB 5 mg/kg/d + POS 400 mg BID	Primary, disseminated IM (*R. microsporus*)	Survived	Mild chronic renal insufficiency	N	
Roux, 2010[88]	Case report	NR	13F metastatic osteosarcoma	1	L-AmB 10 mg/kg/d + POS; CAS added 1 month later	Primary, disseminated IM (*Absidia corymbifera*)	Survived	NR	Y	POS dose not specified
Ogawa, 2012[89]	Case report	NR	70M diabetes	1	L-AmB 10 mg/kg/d + MFG 6 mg/kg/d	Primary, invasive fungal sinusitis (*R. oryzae*)	Survived	NR	N	
Bourke, 2012[90]	Case report	2007	53M heart transplant	1	L-AmB 7.5 mg/kg/d + POS 200 mg QID	Salvage, invasive fungal sinusitis *(Rhizopus* spp.)	Survived	Acute kidney injury	N	

Guymer, 2013[91]	Case report	2011	11F T-ALL	1	L-AmB 10 mg/kg/d + CAS 70 mg/m2 loading dose, then 50 mg/m2/d, then L-AmB 5 mg/kg/d + POS beginning day 82	Primary, disseminated IM (*Lichtheimia corymbifera*)	Survived	NR	Y	Aggressive surgical debridement performed; POS dose not specified
Pagano, 2013[92]	Analysis of SEIFEM and Fungiscope registries	2007-2012	HM	32	ABLC + POS (n=5), L-AmB 3 mg/kg + POS (n=10), L-AmB ≥ 5 mg/kg/d + POS (n=17)	Primary or salvage therapy proven or probable IM	Clinical improvement (CR or PR) in 56% and IM-related death in 28% at median 3 month follow-up	No patients stopped AF treatment due to drug toxicity	NR	Salvage indication for 29 (91%) patients, nearly all started on lipid formulation AmB then POS added; POS dose 800 mg/kg/d in 28 (88%) patients
Klimko, 2014[93]	Case report	2010	53F AML	1	dAmB 1.5 mg/kg/d + CAS	Salvage, musculoskeletal (*Lichtheimia corymbifera*)	Survived	NR	N	
Di Carlo, 2014[94]	Case report	2013	70F diabetes	1	L-AmB 3 mg/kg/d + POS 400 mg BID	Salvage, ROCM	Survived	NR	N	
Kyvernitakis, 2016[95]	Retrospective cohort study, single-center	1994-2014	HM and HCT	106	Monotherapy, n=47: L-AmB < 5 to ≥ 7.5 mg/kg/d (87%) or POS (13%); Combination therapy, n=59: L-AmB + POS (27%), L-AmB + EC (46%), or L-AmB + POS + EC (27%)	Primary therapy of IM	No difference in propensity score-adjusted probability of 6-week mortality between treatment groups (56% monotherapy vs. 60% combination, P = 0.71)	NR	N	Initial combination therapy also had no impact on 6-week survival based on mortality risk score, nor on overall survival at 12 weeks; POS dose not specified
Ville, 2016[96]	Case report	NR	60M kidney transplant	1	L-AmB 10 mg/kg/d + POS 400 mg BID	Primary, disseminated IM (*R. microsporus*)	Died	NR	N	

Al Hassan, 2020[97]	Case series	NR	Diabetes	2	L-AmB 10 mg/kg/d + POS 200 mg QID	Primary, ROCM	Died	NR	N	
Risum, 2020[98]	Retrospective, single-center case series	2012-2019	HM	13	Initial monotherapy, n=4: L-AmB 3-5 mg/kg/d (n=3) or POS (n=1); initial combination therapy, n=9: L-AmB + POS (n=5), L-AmB + POS + TRB (n=1), L-AmB + ISA (n=2), L-AmB + ISA + TRB (n=1)	Primary therapy of proven IM	3-month survival: 7/9 combination patients and 4/4 monotherapy patients; cure achieved in 10 patients (6/9 combination and 4/4 monotherapy)	NR	Y	Nine patients received concomitant topical AF therapy; POS dose 300 mg/d in 5/7 patients; TRB dose 4 mg/kg (n=1) and 1-2 g (n=1)
Salehi, 2020[99]	Case report	NR	35F lymphoma	1	L-AmB 300 mg + POS 200mg QID	Primary, ROCM	Survived	NR	N	
Miller, 2021[100]	Retrospective cohort study, multicenter	2007-2017	HM and HCT	64	Initial monotherapy, n=33: Lipid formulation AmB (Lip-AmB) 5-10 mg/kg/d alone (n=28), POS alone (n=3), ISA alone (n=2); initial combination therapy, n=31: Lip-AmB + POS (n=16) or EC (n=8) or ISA (n=5) or POS + EC (n=2)	Primary therapy proven or probable IM	No difference in treatment failure or 30-day mortality with Lip-AmB alone (64% and 43%, respectively) vs Lip-AmB + POS or ISA (43% and 28%, respectively), P > 0.1	NR	N	

Eubank, 2021[101]	Case report	NR	Liver transplant	3	Case 1: ISA + L-AmB 3 mg/kg/d, then L-AmB + POS 300 mg/d + MFG 150 mg/d; Case 2: L-AmB 3 mg/kg/d + ISA + MFG 150 mg/d, then added terbinafine 500 mg BID and L-AmB irrigation; Case 3: L-AmB 7.5 mg/kg/d + POS 300 mg BID + MFG 150 mg/d	Primary therapy (*Rhizopus* spp.)	Survived	NR	N	Infection sites: musculoskeletal, intra-abdominal, sinus
Beaver, 2021[102]	Case report	NR	47M immunocompetent	1	L-AmB 10 mg/kg/d + ISA x 25 days, then added MFG 100 mg + topical AmB	Primary, sino-orbital IM (*Rhizomucor* spp.)	Survived	Acute kidney injury	N	
Khera, 2021[103]	Case report	NR	6M B-ALL	1	L-AmB 8 mg/kg/d + CAS 50 mg/m2	Salvage, pulmonary	Survived	NR	Y	
Kaushal, 2022[104]	Case series	2017-2019	Diabetes	2	Case 1: L-AmB 10.15 g/d + POS 126.40 g; Case 2: L-AmB 4 g/d + POS 2 g	Salvage, invasive fungal sinusitis, ROCM	Case 1: survived; Case 2: died	none	N	
Loeffen, 2022[105]	Retrospective, single-center case series	2018-2021	HM, pediatric	10 (n=8 proven or probable cases)	Initial therapy: L-AmB 10 mg/kd/d + ISA (n=4), ABLC 7.5-10 mg/kg/d + ISA (n=2), L-AmB 5 mg/kg/d + POS (n=1)	Primary therapy proven, probable, possible IM	Two IM-related deaths, 1 death due to underlying HM, 5 patients alive with 4-22 months of follow-up	5 patients with reversible nephrotoxicity on L-AmB	Y	

ABLC, lipid complex amphotericin B; ALL, acute lymphoblastic leukemia; AML, acute myelogenous leukemia; dAmB, deoxycholate amphotericin B; HM, hematologic malignancy; IM, invasive mucormycosis; ISA, isavuconazole; L-AmB: liposomal amphotericin B; NR: not reported; POS, posaconazole; ROCM, rhinorbital cerebral mucormycosis; SEIFEM: Sorveglianza Epidemiologica Infezioni Fungine Emopatie Maligne; T-ALL, T-cell acute lymphoblastic leukemia; TRB, terbinafine

† Antifungal dosages unless otherwise specified: CAS 70 mg x 1 dose followed by 50 mg/d, ISA 372 mg q 8 h x 6 doses followed by 372 mg/d

References

1. Mikamo H, Ninomiya M, Tamaya T. Tuboovarian abscess caused by Candida glabrata in a febrile neutropenic patient. *J Infect Chemother*. Sep 2003;9(3):257-9. doi:10.1007/s10156-003-0246-6
2. Rex JH, Pappas PG, Karchmer AW, et al. A randomized and blinded multicenter trial of high-dose fluconazole plus placebo versus fluconazole plus amphotericin B as therapy for candidemia and its consequences in nonneutropenic subjects. *Clin Infect Dis*. May 15 2003;36(10):1221-8. doi:10.1086/374850
3. Pelletier R, Alarie I, Lagace R, Walsh TJ. Emergence of disseminated candidiasis caused by Candida krusei during treatment with caspofungin: case report and review of literature. *Med Mycol*. Sep 2005;43(6):559-64. doi:10.1080/13693780500220415
4. Sim JP, Kho BC, Liu HS, Yung R, Chan JC. Candida tropicalis arthritis of the knee in a patient with acute lymphoblastic leukaemia: successful treatment with caspofungin. *Hong Kong Med J*. Apr 2005;11(2):120-3.
5. Lopez-Ciudad V, Castro-Orjales MJ, Leon C, et al. Successful treatment of Candida parapsilosis mural endocarditis with combined caspofungin and voriconazole. *BMC Infect Dis*. Apr 11 2006;6:73. doi:10.1186/1471-2334-6-73
6. Olver WJ, Scott F, Shankland GS. Successful treatment of Candida krusei fungemia with amphotericin B and caspofungin. *Med Mycol*. Nov 2006;44(7):655-7. doi:10.1080/13693780600686929
7. Wong PN, Lo KY, Tong GM, et al. Treatment of fungal peritonitis with a combination of intravenous amphotericin B and oral flucytosine, and delayed catheter replacement in continuous ambulatory peritoneal dialysis. *Perit Dial Int*. Mar-Apr 2008;28(2):155-62.
8. Filippi L, Poggi C, Gozzini E, Meleleo R, Mirabile L, Fiorini P. Neonatal liver abscesses due to Candida infection effectively treated with caspofungin. *Acta Paediatr*. May 2009;98(5):906-9. doi:10.1111/j.1651-2227.2009.01225.x
9. Bland CM, Thomas S. Micafungin plus fluconazole in an infected knee with retained hardware due to Candida albicans. *Ann Pharmacother*. Mar 2009;43(3):528-31. doi:10.1345/aph.1L508
10. Turan O, Ergenekon E, Hirfanoglu IM, et al. Combination antifungal therapy with voriconazole for persistent candidemia in very low birth weight neonates. *Turk J Pediatr*. Jan-Feb 2011;53(1):19-26.
11. Al-Sweih N, Ahmad S, Joseph L, et al. Candida fermentati as a cause of persistent fungemia in a preterm neonate successfully treated by combination therapy with amphotericin B and caspofungin. *J Clin Microbiol*. Mar 2015;53(3):1038-41. doi:10.1128/JCM.03351-14
12. Reyes HA, Carbajal WH, Valdez LM, Lozada C. Successful medical treatment of infective endocarditis caused by Candida parapsilosis in an immunocompromised patient. *BMJ Case Rep*. Oct 9 2015;2015doi:10.1136/bcr-2015-212128
13. Mpakosi A, Siopi M, Falaina V, et al. Successful therapy of Candida pulcherrima fungemia in a premature newborn with liposomal amphotericin B and micafungin. *Med Mycol Case Rep*. Jun 2016;12:24-7. doi:10.1016/j.mmcr.2016.08.002
14. Al-Sweih N, Ahmad S, Khan S, et al. Persistent Candida conglobata bloodstream infection in a preterm neonate successfully treated by combination therapy with amphotericin B and caspofungin. *J Mycol Med*. Jun 2017;27(2):271-276. doi:10.1016/j.mycmed.2017.01.010
15. Tsakiri S, Aneji C, Domonoske C, Mazur L, Benjamin DK, Jr., Wootton SH. Voriconazole Treatment for an Infant With Intractable Candida glabrata Meningitis. *Pediatr Infect Dis J*. Oct 2018;37(10):999-1001. doi:10.1097/INF.0000000000002073
16. Ahuja T, Fong K, Louie E. Combination antifungal therapy for treatment of Candida parapsilosis prosthetic valve endocarditis and utility of T2Candida Panel(R): A case series. *IDCases*. 2019;15:e00525. doi:10.1016/j.idcr.2019.e00525
17. Keuning MC, Al Moujahid A, Zijlstra WP. Prosthetic Joint Infection of a Revision Knee Arthroplasty with Candida parapsilosis. *Case Rep Orthop*. 2019;2019:3634519. doi:10.1155/2019/3634519

18. Wee LE, Wong CSL, Tan AL, Oh HM. Negative cerebrospinal fluid beta-d-glucan levels as an indicator for treatment cessation ahead of biochemical resolution: A case report of Candida glabrata meningitis. *Med Mycol Case Rep*. Jun 2021;32:47-49. doi:10.1016/j.mmcr.2021.03.003
19. Jacobs SE, Jacobs JL, Dennis EK, et al. Candida auris pan-drug-resistant to four classes of antifungal agents. *Antimicrobial Agents and Chemotherapy*. 2022;66(7):e00053-22.
20. Odysseos G, Mayr U, Bozsaki G, et al. Isavuconazole and Liposomal Amphotericin B as Successful Combination Therapy of Refractory Invasive Candidiasis in a Liver Transplant Recipient: A Case Report and Literature Review. *Mycopathologia*. Feb 2022;187(1):113-120. doi:10.1007/s11046-021-00599-1
21. Sharma S, Samantaray S, Kumar D, et al. Prosthetic valve endocarditis due to Candida parapsilosis - a rare case report. *Access Microbiol*. 2023;5(1)doi:10.1099/acmi.0.000462.v4
22. Brouwer AE, Rajanuwong A, Chierakul W, et al. Combination antifungal therapies for HIV-associated cryptococcal meningitis: A randomised trial. *Lancet*. 2004;363(9423):1764-1767. doi:10.1016/S0140-6736(04)16301-0
23. Bicanic T, Wood R, Meintjes G, et al. High-dose amphotericin B with flucytosine for the treatment of cryptococcal meningitis in HIV-infected patients: a randomized trial. *Clin Infect Dis*. Jul 1 2008;47(1):123-30. doi:10.1086/588792
24. Milefchik E, Leal MA, Haubrich R, et al. Fluconazole alone or combined with flucytosine for the treatment of AIDS-associated cryptococcal meningitis. *Med Mycol*. Jun 2008;46(4):393-5. doi:10.1080/13693780701851695
25. Pappas PG, Chetchotisakd P, Larsen RA, et al. A phase II randomized trial of amphotericin B alone or combined with fluconazole in the treatment of HIV-associated cryptococcal meningitis. *Clin Infect Dis*. Jun 15 2009;48(12):1775-83. doi:10.1086/599112
26. Nussbaum JC, Jackson A, Namarika D, et al. Combination flucytosine and high-dose fluconazole compared with fluconazole monotherapy for the treatment of cryptococcal meningitis: a randomized trial in Malawi. *Clin Infect Dis*. Feb 1 2010;50(3):338-44. doi:10.1086/649861
27. Jackson AT, Nussbaum JC, Phulusa J, et al. A phase II randomized controlled trial adding oral flucytosine to high-dose fluconazole, with short-course amphotericin B, for cryptococcal meningitis. *Aids*. Jul 17 2012;26(11):1363-70. doi:10.1097/QAD.0b013e328354b419
28. Loyse A, Wilson D, Meintjes G, et al. Comparison of the early fungicidal activity of high-dose fluconazole, voriconazole, and flucytosine as second-line drugs given in combination with amphotericin B for the treatment of HIV-associated cryptococcal meningitis. *Clin Infect Dis*. Jan 1 2012;54(1):121-8. doi:10.1093/cid/cir745
29. Muzoora CK, Kabanda T, Ortu G, et al. Short course amphotericin B with high dose fluconazole for HIV-associated cryptococcal meningitis. *J Infect*. Jan 2012;64(1):76-81. doi:10.1016/j.jinf.2011.10.014
30. Day JN, Chau TTH, Wolbers M, et al. Combination Antifungal Therapy for Cryptococcal Meningitis. *New England Journal of Medicine*. 2013;368(14):1291-1302. doi:10.1056/nejmoa1110404
31. Vaidhya SA, Gupta BB, Jha RK, Kumar R. Combination Versus Monotherapy for the Treatment of HIV Associated Cryptococcal Meningitis. *J Clin Diagn Res*. Feb 2015;9(2):Oc14-6. doi:10.7860/jcdr/2015/12360.5601
32. Molloy SF, Kanyama C, Heyderman RS, et al. Antifungal Combinations for Treatment of Cryptococcal Meningitis in Africa. *N Engl J Med*. Mar 15 2018;378(11):1004-1017. doi:10.1056/NEJMoa1710922
33. Jandhyala D, Tan EM, Stahr DC, Sohail MR. Disseminated Cryptococcus neoformans infection in a left ventricular assist device recipient. *BMJ Case Rep*. Apr 8 2019;12(4)doi:10.1136/bcr-2018-228283
34. Jarvis JN, Leeme TB, Molefi M, et al. Short-course High-dose Liposomal Amphotericin B for Human Immunodeficiency Virus-associated Cryptococcal Meningitis: A Phase 2 Randomized Controlled Trial. *Clin Infect Dis*. Jan 18 2019;68(3):393-401. doi:10.1093/cid/ciy515
35. Li Z, Liu Y, Chong Y, et al. Fluconazole plus flucytosine is a good alternative therapy for non-HIV and non-transplant-associated cryptococcal meningitis: A retrospective cohort study. *Mycoses*. Aug 2019;62(8):686-691. doi:10.1111/myc.12944
36. Jarvis JN, Lawrence DS, Meya DB, et al. Single-Dose Liposomal Amphotericin B Treatment for Cryptococcal Meningitis. *N Engl J Med*. Mar 24 2022;386(12):1109-1120. doi:10.1056/NEJMoa2111904

37. Takazono T, Hidaka Y, Morimoto S, et al. Comparison of liposomal amphotericin B alone and in combination with flucytosine in the treatment of non-HIV Cryptococcal meningitis: A nationwide observational study. *Mycoses*. Sep 2022;65(9):897-902. doi:10.1111/myc.13493
38. Zhao HZ, Cheng JH, Zhou LH, et al. Induction therapy with high-dose fluconazole plus flucytosine for human immunodeficiency virus-uninfected cryptococcal meningitis patients: Feasible or not? *Mycoses*. Jan 2023;66(1):59-68. doi:10.1111/myc.13528
39. Segal BH, Herbrecht R, Stevens DA, et al. Defining responses to therapy and study outcomes in clinical trials of invasive fungal diseases: Mycoses Study Group and European Organization for Research and Treatment of Cancer consensus criteria. *Clin Infect Dis*. Sep 1 2008;47(5):674-83. doi:10.1086/590566
40. Elanjikal Z, Sörensen J, Schmidt H, et al. Combination therapy with caspofungin and liposomal amphotericin B for invasive aspergillosis. *Pediatr Infect Dis J*. Jul 2003;22(7):653-6. doi:10.1097/01.inf.0000073060.20277.29
41. Lujber L, Gerlinger I, Kuncz A, Pytel J. Combination therapy for chronic invasive rhinocerebral aspergillosis in a clinically immunocompetent patient. *Curr Ther Res Clin Exp*. Jul 2003;64(7):473-83. doi:10.1016/s0011-393x(03)00111-5
42. Kontoyiannis DP, Hachem R, Lewis RE, et al. Efficacy and toxicity of caspofungin in combination with liposomal amphotericin B as primary or salvage treatment of invasive aspergillosis in patients with hematologic malignancies. *Cancer*. Jul 15 2003;98(2):292-9. doi:10.1002/cncr.11479
43. Damaj G, Ivanov V, Le Brigand B, et al. Rapid improvement of disseminated aspergillosis with caspofungin/voriconazole combination in an adult leukemic patient. *Ann Hematol*. Jun 2004;83(6):390-3. doi:10.1007/s00277-003-0792-0
44. Marr KA, Boeckh M, Carter RA, Hyung WK, Corey L. Combination antifungal therapy for invasive aspergillosis. *Clinical Infectious Diseases*. 2004;39(6):797-802. doi:10.1086/423380
45. Rosen-Wolff A, Koch A, Friedrich W, Hahn G, Gahr M, Roesler J. Successful elimination of an invasive Aspergillus nidulans lung infection by voriconazole after failure of a combination of caspofungin and liposomal amphotericin B in a boy with chronic granulomatous disease. *Pediatr Infect Dis J*. Jun 2004;23(6):584-6. doi:10.1097/01.inf.0000130741.01940.ff
46. Castagnola E, Machetti M, Cappelli B, et al. Caspofungin associated with liposomal amphotericin B or voriconazole for treatment of refractory fungal pneumonia in children with acute leukaemia or undergoing allogeneic bone marrow transplant. *Clin Microbiol Infect*. Mar 2004;10(3):255-7. doi:10.1111/j.1198-743x.2004.00837.x
47. Elzi L, Laifer G, Bremerich J, Vosbeck J, Mayr M. Invasive apergillosis with myocardial involvement after kidney transplantation. *Nephrol Dial Transplant*. Mar 2005;20(3):631-4. doi:10.1093/ndt/gfh625
48. Schuster F, Moelter C, Schmid I, et al. Successful antifungal combination therapy with voriconazole and caspofungin. *Pediatr Blood Cancer*. Jun 15 2005;44(7):682-5. doi:10.1002/pbc.20302
49. Kontoyiannis DP, Boktour M, Hanna H, Torres HA, Hachem R, Raad, II. Itraconazole added to a lipid formulation of amphotericin B does not improve outcome of primary treatment of invasive aspergillosis. *Cancer*. Jun 1 2005;103(11):2334-7. doi:10.1002/cncr.21057
50. Pancham S, Hemmaway C, New H, et al. Caspofungin for invasive fungal infections: combination treatment with liposomal amphotericin B in children undergoing hemopoietic stem cell transplantation. *Pediatr Transplant*. Apr 2005;9(2):254-7. doi:10.1111/j.1399-3046.2005.00261.x
51. Lehrnbecher T, Becker M, Schwabe D, et al. Primary intestinal aspergillosis after high-dose chemotherapy and autologous stem cell rescue. *Pediatr Infect Dis J*. May 2006;25(5):465-6. doi:10.1097/01.inf.0000217475.83393.f6
52. Denning DW, Marr KA, Lau WM, et al. Micafungin (FK463), alone or in combination with other systemic antifungal agents, for the treatment of acute invasive aspergillosis. *J Infect*. Nov 2006;53(5):337-49. doi:10.1016/j.jinf.2006.03.003
53. Maertens J, Glasmacher A, Herbrecht R, et al. Multicenter, noncomparative study of caspofungin in combination with other antifungals as salvage therapy in adults with invasive aspergillosis. *Cancer*. Dec 15 2006;107(12):2888-97. doi:10.1002/cncr.22348
54. Singh N, Limaye AP, Forrest G, et al. Combination of voriconazole and caspofungin as primary therapy for invasive aspergillosis in solid organ transplant recipients: a prospective, multicenter, observational study. *Transplantation*. 2006;81(3):320-6. doi:10.1097/01.tp.0000202421.94822.f7
55. Cesaro S, Giacchino M, Locatelli F, et al. Safety and efficacy of a caspofungin-based combination therapy for treatment of proven or probable aspergillosis in pediatric hematological patients. *BMC Infect Dis*. Apr 18 2007;7:28. doi:10.1186/1471-2334-7-28

56. Caillot D, Thiébaut A, Herbrecht R, et al. Liposomal amphotericin B in combination with caspofungin for invasive aspergillosis in patients with hematologic malignancies: a randomized pilot study (Combistrat trial). *Cancer*. Dec 15 2007;110(12):2740-6. doi:10.1002/cncr.23109
57. Meyer M, Waldvogel S, Chalandon Y, Bongiovanni M, Pache JC, Van Delden C. Breakthrough invasive pulmonary aspergillosis despite empirical voriconazole therapy for febrile neutropenia: case report and review of the literature. *Scand J Infect Dis*. 2007;39(8):731-3. doi:10.1080/00365540701199857
58. Gubler C, Wildi SM, Imhof A, Schneemann M, Mullhaupt B. Disseminated invasive aspergillosis with cerebral involvement successfully treated with caspofungin and voriconazole. *Infection*. Oct 2007;35(5):364-6. doi:10.1007/s15010-007-6165-1
59. Panda NK, Saravanan K, Chakrabarti A. Combination antifungal therapy for invasive aspergillosis: can it replace high-risk surgery at the skull base? *Am J Otolaryngol*. Jan-Feb 2008;29(1):24-30. doi:10.1016/j.amjoto.2006.12.004
60. Raad, II, Hanna HA, Boktour M, et al. Novel antifungal agents as salvage therapy for invasive aspergillosis in patients with hematologic malignancies: posaconazole compared with high-dose lipid formulations of amphotericin B alone or in combination with caspofungin. *Leukemia*. Mar 2008;22(3):496-503. doi:10.1038/sj.leu.2405065
61. Saijo T, Izumikawa K, Takazono T, et al. A case of Legionella pneumophila pneumonia followed by invasive aspergillosis. *Jpn J Infect Dis*. Sep 2008;61(5):379-81.
62. Matsuda T, Koreeda Y, Mataki H, Taira T, Noma S, Higashimoto I. A case of Aspergillus empyema successfully treated with combination therapy of voriconazole and micafungin: excellent penetration of voriconazole and micafungin into pleural fluid. *Intern Med*. 2010;49(12):1163-9. doi:10.2169/internalmedicine.49.2860
63. Yamada R, Horikawa K, Ishihara S, et al. Successful treatment of Aspergillus liver abscesses in a patient with acute monoblastic leukemia using combination antifungal therapy including micafungin as a key drug. *Int J Hematol*. May 2010;91(4):711-5. doi:10.1007/s12185-010-0556-2
64. Aoki T, Miyamoto T, Mori Y, et al. Successful allogeneic stem cell transplantation in two patients with acute myelogenous leukaemia and invasive aspergillosis by antifungal combination therapy. *Mycoses*. Jul 2011;54(4):e255-9. doi:10.1111/j.1439-0507.2010.01858.x
65. Beiras-Fernandez A, Bigdeli AK, Nickel T, et al. Combination antifungal therapy for invasive pulmonary aspergillosis in a heart transplant recipient. *Exp Clin Transplant*. Aug 2011;9(4):279-83.
66. Kerl K, Koch B, Fegeler W, Rossig C, Ehlert K, Groll AH. Catheter-associated aspergillosis of the chest wall following allogeneic hematopoietic stem cell transplantation. *Transpl Infect Dis*. Apr 2011;13(2):182-5. doi:10.1111/j.1399-3062.2010.00559.x
67. Lazaro M, Ramos A, Ussetti P, et al. Aspergillus endocarditis in lung transplant recipients: case report and literature review. *Transpl Infect Dis*. Apr 2011;13(2):186-91. doi:10.1111/j.1399-3062.2010.00589.x
68. Dupouey J, Faucher B, Normand AC, et al. Late post-operative Aspergillus flavus endocarditis: Demonstration of a six years incubation period using microsatellite typing. *Med Mycol Case Rep*. 2012;1(1):29-31. doi:10.1016/j.mmcr.2012.05.001
69. Döring M, Zierl A, Mezger M, Lang P, Handgretinger R, Müller I. Eradication of pulmonary aspergillosis in an adolescent patient undergoing three allogeneic stem cell transplantations for acute lymphoblastic leukemia. *Case Rep Transplant*. 2012;2012:672923. doi:10.1155/2012/672923
70. Rojas R, Molina JR, Jarque I, et al. Outcome of Antifungal Combination Therapy for Invasive Mold Infections in Hematological Patients is Independent of the Chosen Combination. *Mediterr J Hematol Infect Dis*. 2012;4(1):e2012011. doi:10.4084/MJHID.2012.011
71. Pillai J, Mubeen M, Chaudhari A, Dalmia B. Aspergillus pacemaker lead endocarditis. *Asian Cardiovasc Thorac Ann*. Apr 2013;21(2):211-4. doi:10.1177/0218492312450448
72. Demir T, Ergenoglu MU, Ekinci A, Tanrikulu N, Sahin M, Demirsoy E. Aspergillus flavus endocarditis of the native mitral valve in a bone marrow transplant patient. *Am J Case Rep*. Jan 21 2015;16:25-30. doi:10.12659/AJCR.892428
73. Marr KA, Schlamm HT, Herbrecht R, et al. Combination antifungal therapy for invasive aspergillosis a randomized trial. *Annals of Internal Medicine*. 2015;162(2):81-89. doi:10.7326/M13-2508

74. Raad, II, Zakhem AE, Helou GE, Jiang Y, Kontoyiannis DP, Hachem R. Clinical experience of the use of voriconazole, caspofungin or the combination in primary and salvage therapy of invasive aspergillosis in haematological malignancies. *Int J Antimicrob Agents*. Mar 2015;45(3):283-8. doi:10.1016/j.ijantimicag.2014.08.012
75. Rofaiel R, Turkistani Y, McCarty D, Hosseini-Moghaddam SM. Fungal mobile mass on echocardiogram: native mitral valve Aspergillus fumigatus endocarditis. *BMJ Case Rep*. Dec 8 2016;2016doi:10.1136/bcr-2016-217281
76. Nicolè S, Lanzafame M, Cazzadori A, et al. Successful Antifungal Combination Therapy and Surgical Approach for Aspergillus fumigatus Suppurative Thyroiditis Associated with Thyrotoxicosis and Review of Published Reports. *Mycopathologia*. Oct 2017;182(9-10):839-845. doi:10.1007/s11046-017-0145-5
77. Chen TK, Groncy PK, Javahery R, et al. Successful treatment of Aspergillus ventriculitis through voriconazole adaptive pharmacotherapy, immunomodulation, and therapeutic monitoring of cerebrospinal fluid (1→3)-β-D-glucan. *Med Mycol*. Jan 1 2017;55(1):109-117. doi:10.1093/mmy/myw118
78. Lee DG, Lee HJ, Yan JL, Lin SS, Aram JA. Efficacy and safety of combination antifungal therapy in Korean haematological patients with invasive aspergillosis. *Mycoses*. Oct 2019;62(10):969-978. doi:10.1111/myc.12972
79. Calvo-Cano A, Garduño-Eseverri E, Alastruey-Izquierdo A, Hernández-Gallego R, Martínez-Gallardo R, Rodríguez-Vidigal FF. Successful treatment of invasive aspergillosis caused by Aspergillus parafelis in a kidney transplant recipient. *Med Mycol Case Rep*. Dec 2020;30:35-38. doi:10.1016/j.mmcr.2020.10.001
80. Furudate A, Hirose S, Abe K, et al. Infantile Aspergillus fumigatus ventriculitis successfully treated with monitoring of plasma and cerebrospinal fluid voriconazole concentration level. *J Infect Chemother*. Jan 2020;26(1):132-135. doi:10.1016/j.jiac.2019.06.011
81. Lennard K, Bannan A, Grant P, Post J. Potential benefit of combination antifungal therapy in Aspergillus endocarditis. *BMJ Case Rep*. Jun 11 2020;13(6)doi:10.1136/bcr-2019-234008
82. Mendoza MA, Anderson A, Morris MI, et al. Successful Treatment of Invasive Fungal Infection Due to Highly Resistant Aspergillus calidoustus in an Allogeneic Hematopoietic Cell Transplant Recipient. *Mycopathologia*. Apr 2020;185(2):399-403. doi:10.1007/s11046-019-00423-x
83. Camargo JF, Jabr R, Anderson AD, et al. Successful Treatment of Disseminated Disease Due to Highly Resistant Aspergillus calidoustus with a Novel Antifungal Therapy. *Antimicrob Agents Chemother*. Mar 15 2022;66(3):e0220621. doi:10.1128/aac.02206-21
84. Nakaya Y, Nakashima Y, Harada N, et al. Successful treatment of proven coronavirus disease 2019-associated pulmonary aspergillosis with liposomal amphotericin B in a patient with bronchiolitis obliterans syndrome after allogeneic hematopoietic stem cell transplantation. *J Infect Chemother*. Feb 2023;29(2):223-227. doi:10.1016/j.jiac.2022.10.020
85. Rickerts V, Atta J, Herrmann S, et al. Successful treatment of disseminated mucormycosis with a combination of liposomal amphotericin B and posaconazole in a patient with acute myeloid leukaemia. *Mycoses*. 2006;49 Suppl 1:27-30. doi:10.1111/j.1439-0507.2006.01299.x
86. Reed C, Bryant R, Ibrahim AS, et al. Combination polyene-caspofungin treatment of rhino-orbital-cerebral mucormycosis. *Clin Infect Dis*. Aug 1 2008;47(3):364-71. doi:10.1086/589857
87. Lebeau O, Van Delden C, Garbino J, et al. Disseminated Rhizopus microsporus infection cured by salvage allogeneic hematopoietic stem cell transplantation, antifungal combination therapy, and surgical resection. *Transpl Infect Dis*. Jun 2010;12(3):269-72. doi:10.1111/j.1399-3062.2009.00484.x
88. Roux BG, Méchinaud F, Gay-Andrieu F, et al. Successful triple combination therapy of disseminated absidia corymbifera infection in an adolescent with osteosarcoma. *J Pediatr Hematol Oncol*. Mar 2010;32(2):131-3. doi:10.1097/MPH.0b013e3181ca0dcf
89. Ogawa T, Takezawa K, Tojima I, et al. Successful treatment of rhino-orbital mucormycosis by a new combination therapy with liposomal amphotericin B and micafungin. *Auris Nasus Larynx*. Apr 2012;39(2):224-8. doi:10.1016/j.anl.2011.03.006
90. Bourke P, Castro P, Rabagliati R, et al. Zygomycosis over-infection during voriconazole therapy for aspergillosis in a heart transplant patient, successfully treated with liposomal amphotericin and posaconazole. *Transpl Infect Dis*. Oct 2012;14(5):E56-9. doi:10.1111/j.1399-3062.2012.00772.x
91. Guymer C, Khurana S Fau - Suppiah R, Suppiah R Fau - Hennessey I, Hennessey I Fau - Cooper C, Cooper C. Successful treatment of disseminated mucormycosis in a neutropenic patient with T-cell acute lymphoblastic leukaemia. LID - 10.1136/bcr-2013-009577 [doi] LID - bcr2013009577. 2013;(1757-790X (Electronic))

92. Pagano L, Cornely OA, Busca A, et al. Combined antifungal approach for the treatment of invasive mucormycosis in patients with hematologic diseases: a report from the SEIFEM and FUNGISCOPE registries. *Haematologica*. Oct 2013;98(10):e127-30. doi:10.3324/haematol.2012.083063
93. Klimko NN, Khostelidi SN, Volkova AG, et al. Mucormycosis in haematological patients: case report and results of prospective study in Saint Petersburg, Russia. *Mycoses*. Dec 2014;57 Suppl 3:91-6. doi:10.1111/myc.12247
94. Di Carlo P, Pirrello R, Guadagnino G, et al. Multimodal surgical and medical treatment for extensive rhinocerebral mucormycosis in an elderly diabetic patient: a case report and literature review. *Case Rep Med*. 2014;2014:527062. doi:10.1155/2014/527062
95. Kyvernitakis A, Torres HA, Jiang Y, Chamilos G, Lewis RE, Kontoyiannis DP. Initial use of combination treatment does not impact survival of 106 patients with haematologic malignancies and mucormycosis: a propensity score analysis. *Clinical Microbiology and Infection*. 2016;22(9):811.e1-811.e8. doi:10.1016/j.cmi.2016.03.029
96. Ville S, Talarmin JP, Gaultier-Lintia A, et al. Disseminated Mucormycosis With Cerebral Involvement Owing to Rhizopus Microsporus in a Kidney Recipient Treated With Combined Liposomal Amphotericin B and Posaconazole Therapy. *Exp Clin Transplant*. Feb 2016;14(1):96-9. doi:10.6002/ect.2014.0093
97. Al Hassan F, Aljahli M, Molani F, Almomen A. Rhino-orbito-cerebral mucormycosis in patients with uncontrolled diabetes: A case series. *Int J Surg Case Rep*. 2020;73:324-327. doi:10.1016/j.ijscr.2020.07.011
98. Risum M, Helweg-Larsen J, Petersen SL, et al. Introduction of a Comprehensive Diagnostic and Interdisciplinary Management Approach in Haematological Patients with Mucormycosis: A Pre and Post-Intervention Analysis. *J Fungi (Basel)*. Nov 8 2020;6(4)doi:10.3390/jof6040268
99. Salehi M, Shahi F, Rizvi FS, et al. Combination antifungal therapy without craniotomy in an immunocompromised patient with rhino-orbito-cerebral mucormycosis: A case report. *Caspian J Intern Med*. 2020;11(2):227-230. doi:10.22088/cjim.11.2.227
100. Miller MA, Molina KC, Gutman JA, et al. Mucormycosis in Hematopoietic Cell Transplant Recipients and in Patients with Hematological Malignancies in the Era of New Antifungal Agents. *Open Forum Infectious Diseases*. 2021;8(2):1-6. doi:10.1093/ofid/ofaa646
101. Eubank TA, Mobley CM, Moaddab M, et al. Successful Treatment of Invasive Mucormycosis in Orthotopic Liver Transplant Population. *Case Rep Transplant*. 2021;2021:8667589. doi:10.1155/2021/8667589
102. Beaver R, Garza B, Vallabhaneni H, Cahuayme-Zuniga L, Midturi J, LaDow T. Use of topical amphotericin in a case of refractory sino-orbital angioinvasive mucormycosis. *Med Mycol Case Rep*. Sep 2021;33:21-25. doi:10.1016/j.mmcr.2021.06.003
103. Khera S, Singh V, Pattanayak S. Favourable outcome in a child with acute lymphoblastic leukaemia and pulmonary mucormycosis managed with combination antifungal therapy of liposomal amphotericin B and caspofungin. *BMJ Case Rep*. Oct 19 2021;14(10)doi:10.1136/bcr-2021-245329
104. Kaushal D, Rajan N, Soni K, et al. Reducing mortality in mucormycosis of the head and neck in diabetic patients: A CARE case series. *Eur Ann Otorhinolaryngol Head Neck Dis*. May 2022;139(3):146-152. doi:10.1016/j.anorl.2021.06.015
105. Loeffen YGT, Scharloo F, Goemans BF, et al. Mucormycosis in Children With Hematologic Malignancies: A Case Series and Review of the Literature. *Pediatr Infect Dis J*. Sep 1 2022;41(9):e369-e376. doi:10.1097/INF.0000000000003608

Supplementary Figure 1. Literature analysis of publications pertaining to laboratory testing of antifungal drugs in combinations against yeasts and molds

Laboratory Methods

Search Criteria: PubMed (1984-2024)

Generic Search Terms (All fields)	
Activity (Activities)	6,327,959
Testing	3,371,750
Combination	2,685,343
In vitro	1,927,465
Susceptibility	642,043
Antifungal	232,801
Antifungal combination	32,729
Broth dilution	5,002
Antifungal combination testing	6,168
In vitro antifungal combination	5,269
In vitro activity antifungal combination	3,293
Antifungal combination susceptibility	1,873
Antifungal combination susceptibility testing	1,117
Antifungal combination testing broth dilution	76
Antifungal combination susceptibility testing broth dilution	37
In vitro activity antifungal combination broth dilution	29

Google scholar

Antifungal combination testing (exact phrase)	50 (10)

76+37+29 publications in English examined for the inclusion of approved antifungal drugs and QC strains = 22 studies
